# Long‐Term Dietary Restriction Has a Strong and Positive Effect on Both Hepatic and Peripheral Insulin Sensitivity, in an Age‐ and Diet‐Dependent Manner

**DOI:** 10.1111/acel.70285

**Published:** 2025-11-10

**Authors:** Joel C. Fisher, Aaffien C. Reijne, Kaja Hribar, Marcel A. Vieira‐Lara, Alzbeta Talarovicova, Dirk‐Jan Reijngoud, David D. van Niekerk, Jacky L. Snoep, Gertjan van Dijk, Barbara M. Bakker

**Affiliations:** ^1^ Department of Biochemistry Stellenbosch University Stellenbosch South Africa; ^2^ Groningen Institute for Evolutionary Life Sciences, Department of Behavioral Neuroscience University of Groningen Groningen the Netherlands; ^3^ Laboratory of Pediatrics, University Medical Center Groningen University of Groningen Groningen the Netherlands; ^4^ Department of Biotechnology Delft University of Technology Delft HZ the Netherlands

**Keywords:** ageing, diet, dietary restriction, glucose homeostasis, insulin resistance, insulin sensitivity

## Abstract

Dietary restriction (DR) improves insulin sensitivity, however, it has not been tested in long‐term interventions and with diet type as variable. Therefore, we exposed mice to either a low‐fat (LF) or high‐fat‐sucrose (HFS) diet, either fed *ad libitum* (AL) or in a DR regimen from weaning till 2 years of age. Using an oral glucose tolerance test with [6,6‐^2^H_2_]‐labelled glucose, we found that DR markedly reduced plasma insulin concentrations and strongly elevated hepatic and peripheral insulin sensitivity when compared to AL cohorts. These effects of DR, however, appeared to depend on diet and age, with stable increases in hepatic and peripheral insulin sensitivities across all ages in the LF condition, while these became clearly less elevated in the HFS condition with advancing age.

## Graphical abstract

The effect of life‐long dietary restriction was investigated alongside a low‐fat or high‐fat‐sucrose diet on hepatic and peripheral insulin sensitivity. Dietary restriction improves both hepatic and peripheral insulin sensitivity in an age and diet‐dependent manner.

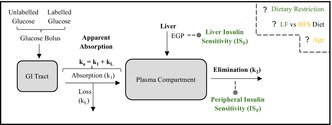

## 1 Introduction

The effects of dietary restriction (DR) on glucose homeostasis and associated health factors have been studied extensively for short‐term interventions (e.g., de Souza et al. 2021; Flanagan et al. 2020; Hofer et al. 2022; Kebbe et al. 2021; Martín et al. 2021; Most and Redman 2020; Napoleão et al. 2021). Interestingly, mice on a 4‐week DR regimen and eating a high‐fat (HF) diet improved whole‐body insulin sensitivity (IS), albeit not to levels observed in a low‐fat (LF) diet control cohort (Zhang et al. 2022). DR studies on liver versus peripheral (skeletal muscle) IS are lacking, except for an 11 week spanning DR study in obese humans by Kirk and colleagues (Kirk et al. 2009), where DR improved both hepatic and peripheral IS. To assess long term consequences of DR in relation to diet, we investigated in mice lifelong interactions between diet type and DR (vs. *ad libitum*, AL) on IS at the peripheral and hepatic tissue level. We observed a dramatic improvement by DR in both hepatic and peripheral IS, which was diet‐ and age‐dependent.

## 2 Research Design and Methods

Male C57BL6/JOlaHsd mice were fed either a LF (6% fat) or HFS (45% fat with added sucrose) diet from weaning onwards, with or without DR as described before (Reijne et al. 2022). The 45% fat content with added sucrose is relevant for comparison to human physiology (Speakman 2019). The HFS restricted cohorts (HFSDR) received 60% of calories consumed by HFSAL cohorts, while LFDR cohorts received either 60% of calories consumed by HFSAL cohorts (equivalent to approximately 70% of calories consumed by LFAL cohorts, LFDR1 at 4 and 15 months), or 60% of calories consumed by the LFAL cohorts (LFDR2 at 9 and 21 months).

**FIGURE 1:**
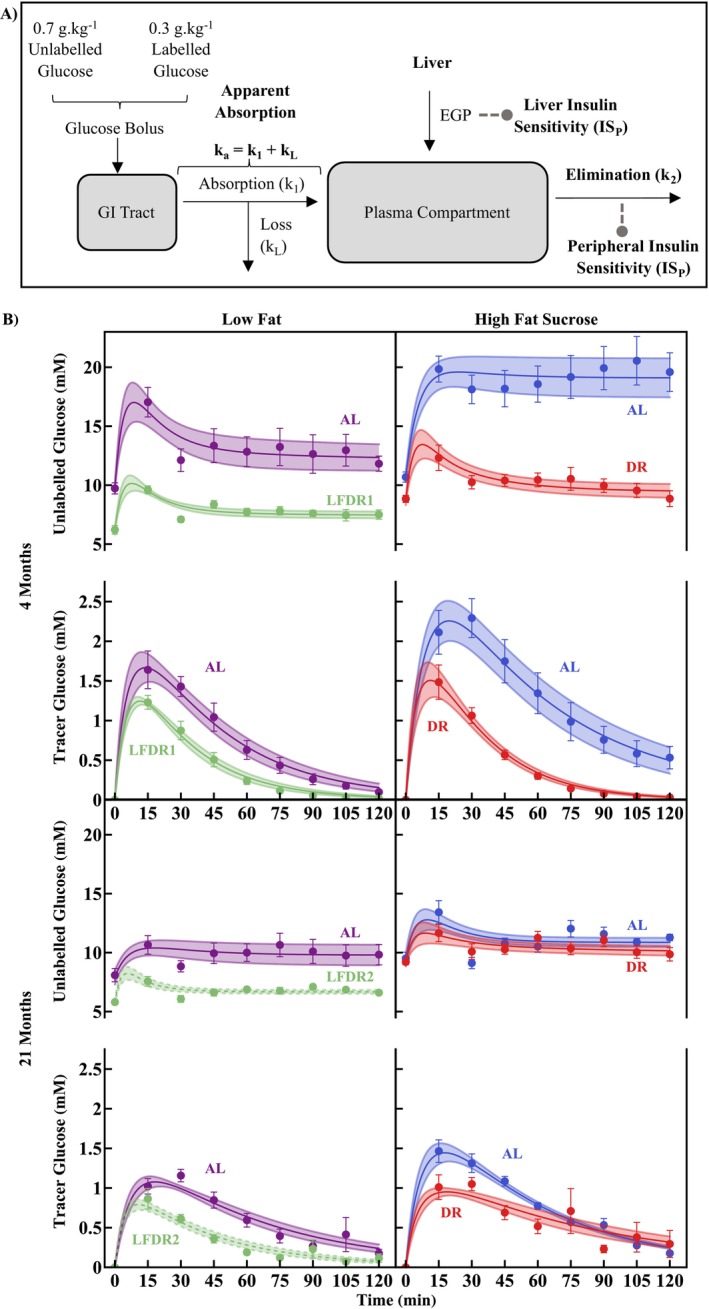
Study design and the effect of diet and dietary provision on plasma glucose dynamics. (A) Schematic representation of the OGTT experimental design and modelling approach. Apparent absorption from GI Tract to Plasma Compartment is shown as k_a_, the sum of the absorption rate constant, k_1_, and the loss rate constant, k_L_. (B) Tracer and Unlabelled glucose time courses for the youngest and oldest cohorts shown in a diet and age paired manner. The coloured bands indicate the mean ± SEM of the individual mouse fits for each cohort. Mean for each timepoint ± SEM are also shown as the datapoints and error bars respectively. Number of mice per cohort: 8.

Mice were fasted for 6 h prior to glucose bolus administration (1 g.(kg BW)^−1^), performed at 4, 9, 15, or 21 months of age. Body weight (BW) of all mice is shown in (Reijne et al. 2022) and for only the OGTT mice in the present study in Figure S1. Despite dosing to BW resulting in obese mice receiving a larger glucose dose for the OGTT, this would not affect the elimination rate constant k_2_ due to its independence of blood glucose concentrations. The bolus consisted of 0.7 g.kg^−1^ unlabelled glucose and 0.3 g.kg^−1^ [6,6‐^2^H_2_]‐glucose (tracer) (Vieira‐Lara et al. 2023). Total blood glucose was measured from the tail vein and blood spots were collected to determine glucose concentrations and tracer enrichment from 0 to 120 min in 15‐min intervals (Van Dijk et al. 2003). Plasma insulin concentration was also measured during the OGTT time course. The glucose isotopologue distribution (m/z 408–412) was corrected for natural occurrence of the tracer isotope in the baseline sample (Vieira‐Lara et al. 2023). The modelling approach (Figure 1A) was based on (Vieira‐Lara et al. 2023), and is outlined in detail in the Supporting Informations section.

Model fits and data visualisation were performed using Wolfram Mathematica. Three‐way (ART‐)ANOVAs were performed in R (4.3.2) or GraphPad Prism (10.4.1). Diet was treated as a factor with 2 levels (LF and HFS), the presence or absence of DR was treated similarly (AL and DR), and Age was treated as having 4 levels (4, 9, 15, 21), except when analysing insulin, peripheral IS (IS_P_) and liver IS (IS_L_) (4,15,21) due to lack of data for the LFDR2 9 month cohort. LFDR1 and LFDR2 were not treated as distinct as they are variations of the same methodology. (ART‐)ANOVA enables determination of whether DR cohorts significantly differ from AL cohorts as a whole and if this is impacted by Diet and/or Age, irrespective of degree of restriction. Where ANOVA assumptions were not met, ART‐ANOVA was performed to test the robustness of the results. (ART‐)ANOVA results are subsequently shown in text and figure captions. All data generated or analysed during this study are included in the published article and its Supporting Information.

## 3 Results

### 3.1 DR Reduced Peak Plasma Glucose and Increases Elimination Rate

Glucose time course data and resulting fits to the kinetic model (Figure 1A,B, all cohorts shown in Figures S3 and S4) highlight the reduction in tracer peak height for plasma glucose concentrations in DR compared to AL (*p*_DR_ = 4.440 × 10^−11^). HFS elevated peak tracer concentration compared to LF (*p*_Diet_ = 6.052 × 10^−9^). Age also had a significant effect (*p*_Age_ = 6.416 × 10^−8^), with peak height generally higher in younger compared to older cohorts.

Estimated elimination rate constants (k_2_) were higher under DR compared to AL (*p*_DR_ = 1.304 × 10^−6^) (Figure S2, from 0.0147 to 0.0297 s^−1^ in AL to 0.015 to 0.0408 s^−1^ in DR), although this effect diminished with age in a diet‐dependent manner (*p*_DRxAge_ = 3.552 × 10^−7^, *p*_DRxDietxAge_ = 0.004624). Parameter k_a_ exhibited limited identifiability (Supporting Informations).

### 3.2 DR Elevated Specific EGP Rate

**FIGURE 2:**
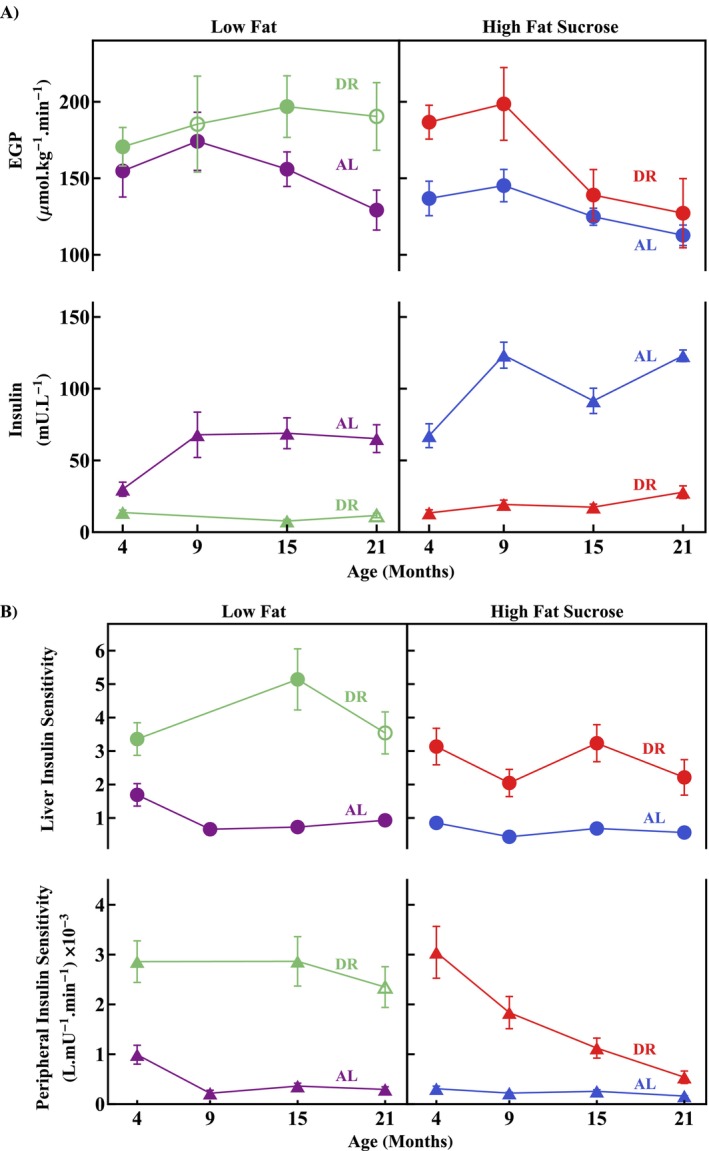
Effect of diet and dietary provision on specific EGP rate, plasma insulin concentration, and liver and peripheral insulin sensitivities. (A) Mean specific EGP (top panel, data shown as discs) calculated from 5 to 120 min ± SEM for each cohort during the OGTT. Mean insulin concentrations (bottom panel, data shown as triangles) ± SEM for each cohort during the OGTT. Insulin concentrations for the LFDR2 9‐month cohort were unavailable. LFDR2 cohorts (receiving 60% of LFAL calories, that is, more restricted than LFDR1 cohorts) are indicated by the open symbols. (B) Liver (top panel, data shown as discs) and peripheral (bottom panel, data shown as triangles) insulin sensitivities for each cohort. Indices are shown as the mean of each cohort ± SEM. IS_L_ is dimensionless as it is normalised to the cohorts of the study. Due to the lack of insulin data for the 9‐month LFDR2 cohort, all 9‐month cohorts were excluded from the 3‐way ANOVA when analysing the insulin data and IS indices. *n* per cohort: EGP: LFAL (4, 15, 21) months = 8, LFAL 9 months = 7. HFSAL (4, 9, 21) months = 8, HFSAL 15 months = 7. LFDR1 (4, 15) months = 8. LFDR2 9 months = 6, LFDR2 21 months = 8. HFSDR (4, 9, 15, 21) months = 8. Insulin: LFAL (4, 15, 21) months = 8, LFAL 9 months = 7. HFSAL (4, 9, 21) months = 8, HFSAL 15 months = 7. LFDR1 4 months = 6, LFDR1 15 months = 8, LFDR2 21 months = 8. HFSDR 4 months = 6, HFSDR 9 months = 9, HFSDR (15, 21) months = 8. IS_L_ (calculated): LFAL (4, 15, 21) months = 8, LFAL 9 months = 7. HFSAL (4, 9, 15, 21) months = 8. LFDR1 4 months = 7, LFDR1 15 months = 8, LFDR2 21 months = 8. HFSDR 4 months = 6, HFSDR (9, 21) months = 9, HFSDR 15 months = 8. IS_P_ (calculated): LFAL (4, 15, 21) months = 8, LFAL 9 months = 7. HFSAL (4, 15, 21) months = 8, HFSAL 9 months = 7. LFDR1 4 months = 7, LFDR 1 15 months = 8, LFDR2 21 months = 8. HFSDR (4) months = 6, HFSDR 9 months = 9, HFSDR (15, 21) months = 8. Significant ART‐ANOVA results for average specific EGP: *p*_DR_ = 0.00054863, *p*_Diet_ = 0.00996772. ART‐ANOVA and ANOVA both confirmed the significant effect of DR and diet, however, age was not determined to be significant by ART‐ANOVA, but was significant according to ANOVA, *p*_Age_ = 0.0283487. This indicates the significance of the effect of age is dependent on the assumptions of the statistical test and is therefore not as robust of a result as the effects of DR and diet. Significant ANOVA results for average insulin: *p*_DR_ < 2.2 × 10^−16^, *p*_Diet_ = 4.483 × 10^−9^, *p*_Age_ = 9.69 × 10^−7^, *p*_DRxDiet_ = 6.225 × 10^−5^, *p*_DRxAge_ = 7.882 × 10^−5^, *p*_DietxAge_ = 0.03717. Significant ANOVA results for IS_L_: *p*_DR_ < 0.0001, *p*_Diet_ < 0.01, *p*_DRxAge_ < 0.05. Significant ANOVA results for IS_P_: *p*_DR_ < 0.0001, *p*_Diet_ < 0.0001, *p*_Age_ < 0.0001, *p*_DRxDietxAge_ < 0.01, *p*_DRxDiet_ < 0.01, *p*_DRxAge_ < 0.05.

When calculating time‐averaged specific EGP, the first 5 min of simulation were excluded since the model most likely overestimated EGP in the initial few minutes. DR elevated average specific EGP compared to AL (*p*_DR_ = 0.00054863, Figure 2A, from 113 to 174 μmol kg^−1^ min^−1^ in AL compared to 127 to 199 μmol kg^−1^ min^−1^ in DR) independent of ageing and diet (*p*_DRxDiet_ = 0.59645910, *p*_DRxAge_ = 0.86760612). See Supporting Information for an interpretation of this apparently counterintuitive effect. HFS lowered average specific EGP compared to LF (*p* = 0.00996772).

### 3.3 DR Reduced Plasma Insulin and Elevates Peripheral and Hepatic IS

Plasma insulin concentrations during OGTTs were strongly reduced by DR compared to AL (*p*_DR_ < 2.2 × 10^−16^, Figure 2A, from 30 to 123 mU L^−1^ in AL to 8 to 28 mU L^−1^ in DR). Age and diet modulated this effect (*p*_DRxDiet_ = 6.255 × 10^−5^, *p*_DRxAge_ = 7.882 × 10^−5^). HFS elevated insulin compared to LF (*p*_Diet_ = 4.483 × 10^−9^). Age also had a significant effect (*p*_Age_ = 9.690 × 10^−7^) with older cohorts generally elevated compared to younger cohorts.

Liver IS (IS_L_) is a quantitative estimate of the response of the liver to insulin secretion using the product of average specific EGP and plasma insulin concentration as an indication of the hepatic response to elevated blood glucose and subsequent increase in plasma insulin (Vieira‐Lara et al. 2023). A smaller product is indicative of greater sensitivity (if insulin is elevated, EGP should be lowered as they are antagonistic). IS_L_ was strongly elevated by DR compared to AL (*p*_DR_ < 0.0001) with age modulating the degree of this effect (*p*_DRxAge_ = 0.0477) (Figure 2B). HFS reduced IS_L_ compared to LF (*p*_Diet_ = 0.0047). LFAL cohorts exhibited improved IS (up to 2‐fold greater) compared to HFSAL, however this improvement was considerably smaller than that of DR compared to AL (Figure 2B). IS_L_ for AL mice was greatest at 4‐months of age.

Contribution of peripheral tissues to reducing plasma glucose in response to insulin secretion (IS_P_) (Vieira‐Lara et al. 2023) is calculated by dividing the elimination rate constant (k_2_, Figure 1A) by the insulin concentration. This quantifies the rate at which glucose is removed from blood in response to insulin, with a larger value indicative of a greater sensitivity. IS_P_ was strongly increased in DR compared to AL cohorts (*p*_DR_ < 0.0001) (Figure 2B). Both Age and Diet modulated the effect of DR compared to AL (*p*_DRxAge_ = 0.0199, *p*_DRxDiet_ = 0.0095, *p*_DRxDietxAge_ = 0.0013) and advanced ageing in combination with an HFS diet almost abolished the effect of DR on IS_P_ (LFDR/HFSDR IS_P_ ratio increased from 0.94 to 4.4 from 4 to 21 months, respectively), with this effect not explained by a loss of muscle (Figure S7). In contrast, IS_P_ remained elevated in all LFDR cohorts. Additionally, LF diet elevated IS_P_ compared to HFS diet (*p*_Diet_ < 0.0001), up to 3‐fold higher IS_P_ for AL cohorts, a much smaller effect than DR compared to AL. Age affected IS_P_ (*p*_Age_ < 0.0001), with older cohorts exhibiting reduced IS_P_ compared to younger cohorts. IS_P_ for AL cohorts was greatest at 4‐months of age.

## 4 Discussion

Among several DR and fasting regimes studied, life‐long DR of 40% applied on a daily basis appears to be most efficacious to extend life span and several associated metabolic and physiological adaptations in mice (Di Francesco et al. 2024). In our study, this protocol in LF feeding mice increased glucose elimination rates with a concomitantly increased specific EGP, and stably reduced plasma insulin concentration constant for all ages, contributing to a rather stably increased IS_P_ and IS_L_. However, HFSDR cohorts exhibited a strong reduction in IS_P_ when comparing 4 to 21 month cohorts, the doubling of the insulin concentration (also seen in the LFAL and HFSAL cohorts) was not sufficient to describe this effect. Instead, the strong reduction in the elimination rate constant, HFSDR (k_2_ 21 month)/(k_2_ 4 month) = 0.37, contributed even more to this observed reduction in IS_P_. Possible mechanisms for preservation of IS in DR groups could run via AMP‐activated protein kinase (AMPK), which was found to be upregulated in liver and white adipose tissue of LFDR as well as HFDR fed mice (Zhang et al. 2022). Since ad libitum feeding of a western style diet down‐regulates AMPK activation (Shiwa et al. 2015), the latter reduction may not be entirely prevented by DR. Unfortunately, we did not assess the time course by which DR treatments and diets may affect AMPK—and its down‐stream targets like mammalian target of rapamycin (Gwinn et al. 2008; Panwar et al. 2023), and how they may have affected insulin‐stimulated glucose uptake in the liver and beyond. A second limitation is the fact that we did not include female mice, although recent literature suggests clear sex‐dependent effects of DR on longevity and glucose homeostasis (Kane et al. 2018). Finally, a general limitation of mouse studies is the low blood volume precluding frequent measurement (cf. Visentin et al. 2015). Although the precise molecular mechanism is unknown, long‐term DR had a strong effect on glucose homeostasis in mice, in particular improving IS, which was age and diet dependent.

## Author Contributions

A.C.R. and A.T. conducted the animal experiments. J.C.F. performed the data analysis and computational modelling, K.H. and M.A.V.‐L. advised about the modelling, J.L.S., D.‐J.R., B.M.B. and G.D. conceptualised the project. B.M.B, G.D., D.D.N., and J.L.S. provided supervision. All authors contributed to writing the manuscript. B.M.B. and G.D. are the guarantors of this work, had access to all data used in the study and guarantee the integrity of the data and subsequent analysis.

## Disclosure

Prior Presentation: Parts of this study were orally presented at the Modelling in the Context of African Health conference 2023, virtual, 12–13 April, and at the International Study Group for Systems Biology 2024 Conference, 9–13 September.

## Conflicts of Interest

The authors declare no conflicts of interest.

## Supporting information


**Figure S1:** Body weights over time for mice included in OGTT experiment. Data shown is the mean of all mice in the respective groups, with the error bars the SEM. Each data point incorporates data from 7–32 mice.

**Figure S2:** Effect of diet and dietary provision on fitted model parameters for apparent glucose absorption and elimination rate constants. Apparent absorption (ka, top panel, data shown as discs) and elimination (k2, bottom panel, data shown as triangles) rate constants obtained from fitting tracer glucose time course data. Means ± SEM are shown for each cohort. The LFDR cohorts have been separated into 2 subcategories based on the different degree of DR applied. LFDR1: LFDR cohorts receiving 60% of HFSAL calories indicated by the closed symbols at 4‐ and 15‐months. LFDR2: LFDR cohorts receiving 60% of LFAL calories (more restrictive), indicated by the open symbols at 9‐ and 21‐months. n per cohort: LFAL (4, 15, 21) months = 8, LFAL 9 months = 7. HFSAL (4, 9, 21) months = 8, HFSAL 15 months = 7. LFDR1 (4, 15) months = 8. LFDR2 9 months = 6, LFDR2 21 months = 8. HFSDR (4, 9, 15, 21) months = 8. Significant ANOVA results for k2: pDR = 1.304 x 10–6, pAge =3.499 x 10–11, pDRxAge =3.552 x 10–7, pDietxAge = 3.064 x 10–5, pDRxDietxAge = 0.004624.

**Figure S3:** Tracer glucose fits for each cohort (age and diet paired). The coloured bands indicate the average ± SEM of the individual mouse fits for each cohort. Mean for each timepoint ± SEM are also shown as the datapoints and error bars respectively. n per cohort: LFAL (4, 15, 21) months = 8, LFAL 9 months = 7. HFSAL (4, 9, 21) months = 8, HFSAL 15 months = 7. LFDR1 (4, 15) months = 8. LFDR2 9 months = 6, LFDR2 21 months = 8. HFSDR (4, 9, 15, 21) months = 8. Significant ANOVA results for peak tracer concentration: pDR = 4.440 x10‐11, pDiet = 6.052 x10‐9, pAge = 6.416 x10‐8.

**Figure S4:** Unlabelled glucose fits for each cohort (age and diet paired). Coloured bands indicate the average ± SEM of the mouse fits. Mean for each timepoint ± SEM are shown as the datapoints and error bars respectively. n per cohort: LFAL (4, 15, 21) months = 8, LFAL 9 months = 7. HFSAL (4, 9, 21) months = 8, HFSAL 15 months = 7. LFDR1 (4, 15) months = 8. LFDR2 9 months = 6, LFDR2 21 months = 8. HFSDR (4, 9, 15, 21) months = 8.

**Figure S5:** Age and diet paired cohort specific EGP (normalised to BW) time courses. The coloured bands indicate the average ± SEM of the individual mouse EGP time courses in each cohort. n per cohort: EGP: LFAL (4, 15, 21) months = 8, LFAL 9 months = 7. HFSAL (4, 9, 21) months = 8, HFSAL 15 months = 7. LFDR1 (4, 15) months = 8. LFDR2 9 months = 6, LFDR2 21 months = 8. HFSDR (4, 9, 15, 21) months = 8. Significant ART‐ANOVA results for steady‐state specific EGP: pDR = 0.00084144, pDiet = 0.00183543, pDietxAge = 0.03077719.

**Figure S6:** Age and diet paired cohort EGP time courses not normalised to BW. The coloured bands indicate the average ± SEM of the individual mouse EGP time courses in each cohort. LFDR1 cohorts are annotated as DR1 and LFDR2 cohorts annotated as DR2 with the time‐courses shown as dashed lines. n per cohort: LFAL (4, 15, 21) months = 8, LFAL 9 months = 7. HFSAL (4, 9, 21) months = 8, HFSAL 15 months = 7. LFDR1 (4, 15) months = 8. LFDR2 9 months = 6, LFDR2 21 months = 8. HFSDR (4, 9, 15, 21) months = 8. Significant ANOVA results for time‐averaged EGP not normalised to BW: pDR = 7.452 x 10–9, pDiet = 0.0104636, pAge = 0.0004068, pDietxAge = 0.0143884. Significant ANOVA results for steady‐state EGP not normalised to BW: pDR = 2.972 x 10–9, pDiet = 0.0110855, pAge = 0.0002709, pDietxAge = 0.0017708.

**Figure S7:** Mouse average quadriceps mass. Mean quadriceps mass (data shown as discs) ± SEM for each cohort. Data shown for 6‐month cohorts are from the quadriceps of 1 leg, not the mean of 2 legs. LFDR2 cohorts (receiving 60% of LFAL calories i.e. more restricted than LFDR1 cohorts) are indicated by the open symbols. n per cohort: LFAL (4, 9, 21) months = 7, LFAL 15 months = 8. HFSAL (4, 9) months = 8, HFSAL (15, 21) months = 7. LFDR1 (4, 15) months = 7, LFDR2 (9, 21) months = 7. HFSDR (4, 21) = 7, HFSDR (9, 15) = 8.

**Figure S8:** Apparent volume of distribution (normalised to BW). Mean apparent volume of distribution normalised to BW ± SEM. LFDR2 cohorts are indicated by the open symbols. n per cohort: LFAL (4, 15, 21) months = 8, LFAL 9 months = 7. HFSAL (4, 9, 21) months = 8, HFSAL 15 months = 7. LFDR1 (4, 15) months = 8. LFDR2 9 months = 6, LFDR2 21 months = 8. HFSDR (4, 9, 15, 21) months = 8. Significant ANOVA results: pDR < 2.2 x 10–16, pDiet < 2.2 x 10–16, pAge < 2.2 x 10–16, pDRxDiet = 0.01729, pDRxAge = 5.597 x 10–12, pDietxAge = 1.5 x 10–12.

**Figure S9:** Apparent volume of distribution (not normalised to BW). Mean apparent volume of distribution (not normalised to BW) ± SEM. LFDR2 cohorts are indicated by the open symbols. n per cohort: LFAL (4, 15, 21) months = 8, LFAL 9 months = 7. HFSAL (4, 9, 21) months = 8, HFSAL 15 months = 7. LFDR1 (4, 15) months = 8. LFDR2 9 months = 6, LFDR2 21 months = 8. HFSDR (4, 9, 15, 21) months = 8. Significant ANOVA results: pDR = 0.002064, pAge < 2.2 x 10–16, pDRxAge = 2.014 x 10–6, pDietxAge = 6.824 x 10–5, pDRxDietxAge = 0.028680.

**Figure S10:** ISL (top panel, data shown as discs) and ISP (bottom panel, data shown as triangles) for the control cohorts analysed by Vieira‐Lara and colleagues (Vieira‐Lara et al. 2023) using their original modelling approach and this adapted approach. ISL is dimensionless as it is normalised to the cohorts of the study. Calculated sample size for each cohort: Original ISL: LFAL 4 months = 9, LFAL (9, 15) months = 7, LFAL 21 months = 8. HFSAL (4, 9) months = 7, HFSAL 15 months = 8, HFSAL 21 months = 6. Adapted ISL: LFAL (4, 15, 21) months = 8, LFAL 9 months = 7. HFSAL (4, 9, 15, 21) months = 8. Original and adapted ISP: LFAL (4, 15, 21) months = 8, LFAL 9 months = 7. HFSAL (4, 15, 21) months = 8, HFSAL 9 months = 7.

**Table S1:** Parameter k2 ANOVA results.
**Table S2:** Peak tracer glucose concentration ANOVA results.
**Table S3:** Average insulin concentration ANOVA results.
**Table S4:** Normalised apparent distribution volume ANOVA results.
**Table S5:** Apparent distribution volume (not normalised to BW) ANOVA results.
**Table S6:** Average specific EGP ANOVA results.
**Table S7:** Average specific EGP ART‐ANOVA results.
**Table S8:** Average EGP (not normalised to BW) ANOVA results.
**Table S9:** Steady‐state specific EGP ART‐ANOVA results.
**Table S10:** Steady‐state EGP (not normalised to BW) ANOVA results.
**Table S11:** ISP ANOVA results.
**Table S12:** ISL ANOVA results.
**Table S13:** Identifiability of ka and k2 parameters (with 95% confidence) expressed as a proportion of mice in each cohort.
**Table S14:** Sample size for each cohort for each metric/measurement.

**Data S1:** InsulinData.

**Data S2:** LabelledGlucose.

**Data S3:** MiceBodyWeightsforVolandEGP(grams).

**Data S4:** MiceBodyWeightsOverTime (grams).

**Data S5:** QuadricepsMass.

**Data S6:** UnlabelledGlucose.

## Data Availability

The data that supports the findings of this study are available in the Supporting Information of this article. Wolfram Mathematica Notebooks used for C selection, fitting, and Figure construction can be found at https://github.com/JoelCF97/Mouse_DR_OGTT_study.
