# Supplementary figures and images for: Long‐Term Dietary Restriction Has a Strong and Positive Effect on Both Hepatic and Peripheral Insulin Sensitivity, in an Age‐ and Diet‐Dependent Manner
Source: Aging Cell. 2025 Nov 10;24(12):e70285. doi: 10.1111/acel.70285 (PMC12686546; doi:10.1111/acel.70285)

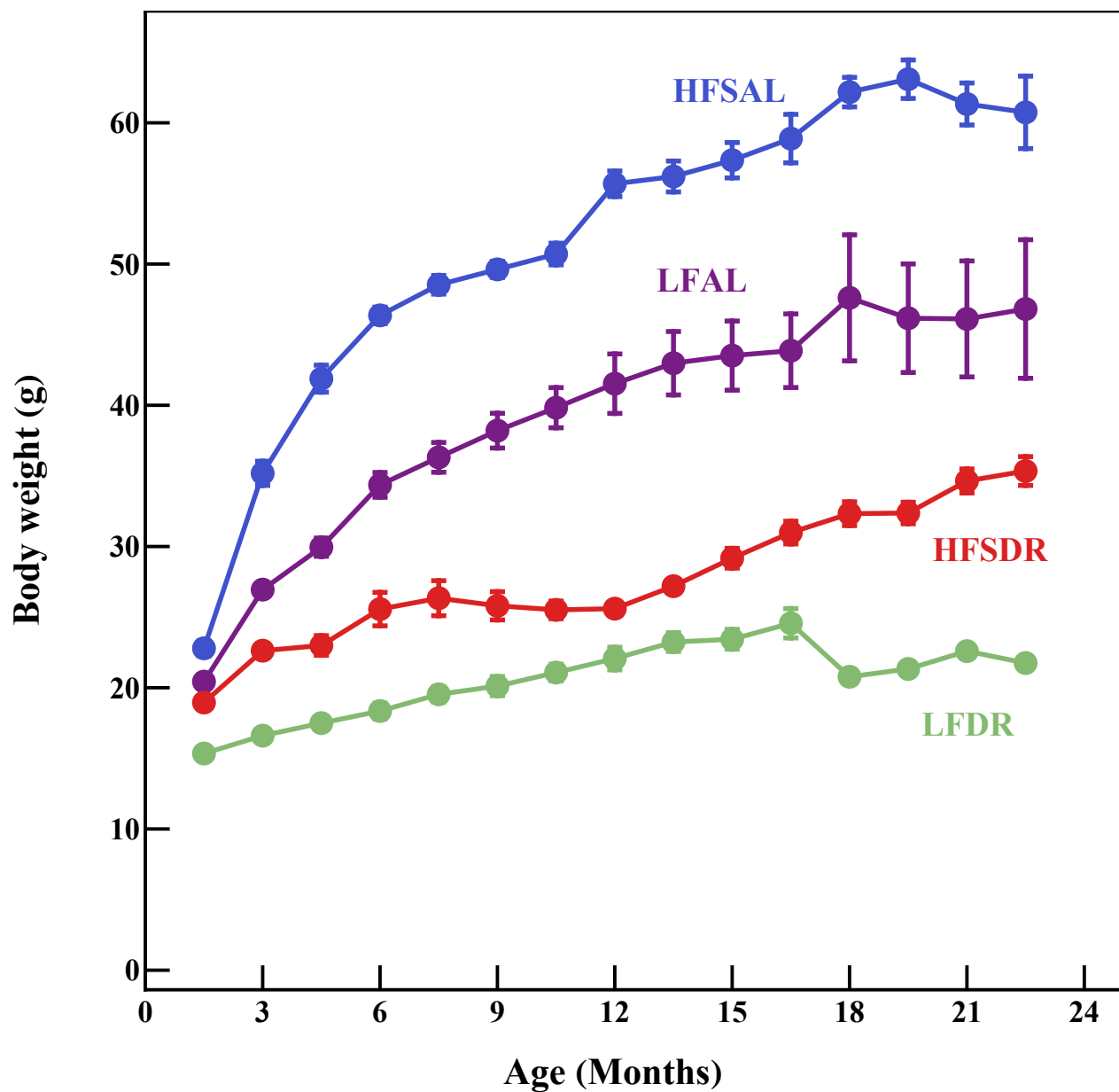

Supplement: Supplementary file 1 — Figure S1: Body weights over time for mice included in OGTT experiment. Data shown is the mean of all mice in the respective groups, with the error bars the SEM. Each data point incorporates data from 7–32 mice. [file ACEL-24-e70285-s010.pdf]

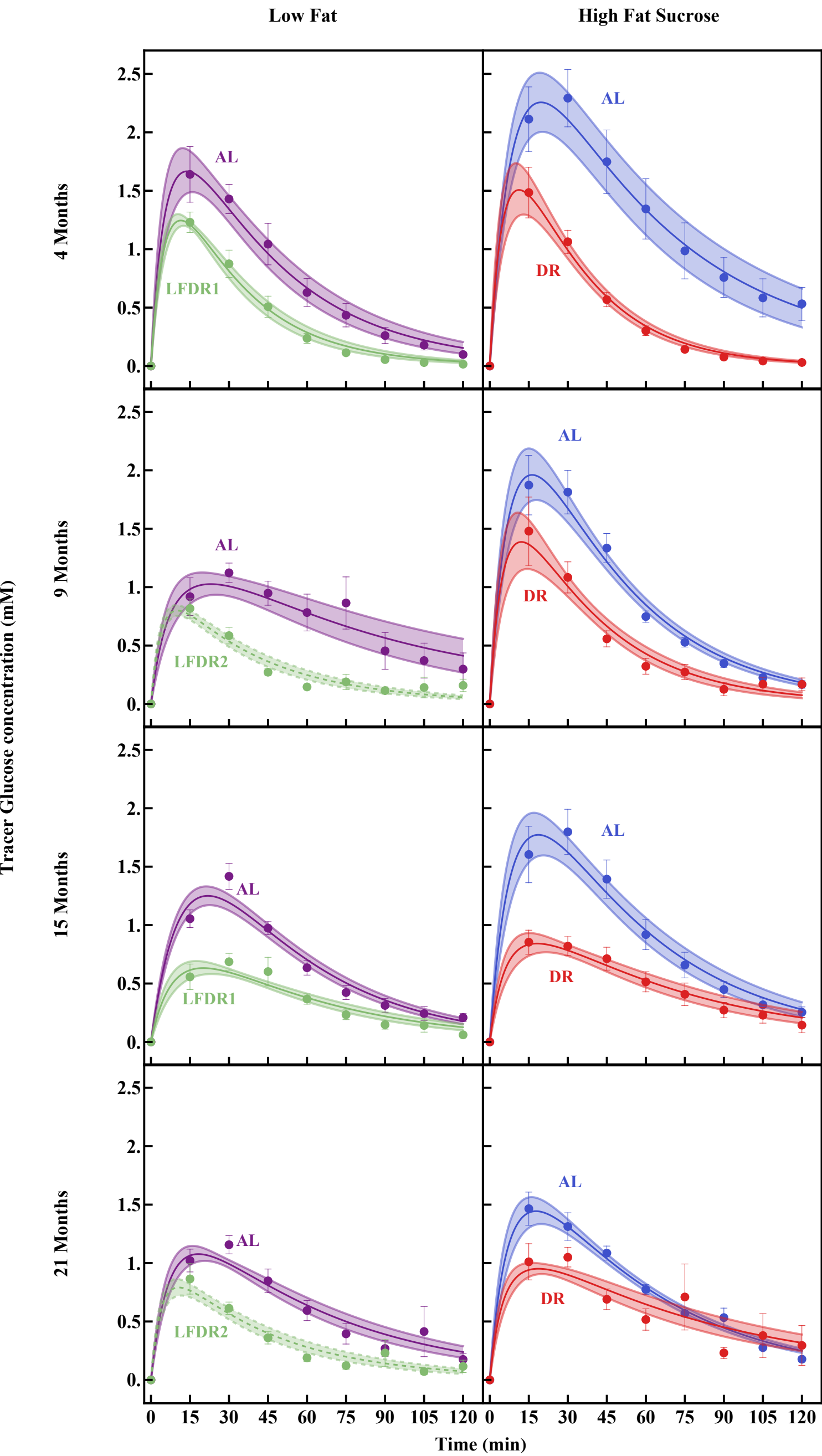

Supplement: Supplementary file 3 — Figure S3: Tracer glucose fits for each cohort (age and diet paired). The coloured bands indicate the average ± SEM of the individual mouse fits for each cohort. Mean for each timepoint ± SEM are also shown as the datapoints and error bars respectively. n per cohort: LFAL (4, 15, 21) months = 8, LFAL 9 months = 7. HFSAL (4, 9, 21) months = 8, HFSAL 15 months = 7. LFDR1 (4, 15) months = 8. LFDR2 9 months = 6, LFDR2 21 months = 8. HFSDR (4, 9, 15, 21) months = 8. Significant ANOVA results for peak tracer concentration: pDR = 4.440 x10‐11, pDiet = 6.052 x10‐9, pAge = 6.416 x10‐8. [file ACEL-24-e70285-s015.pdf]

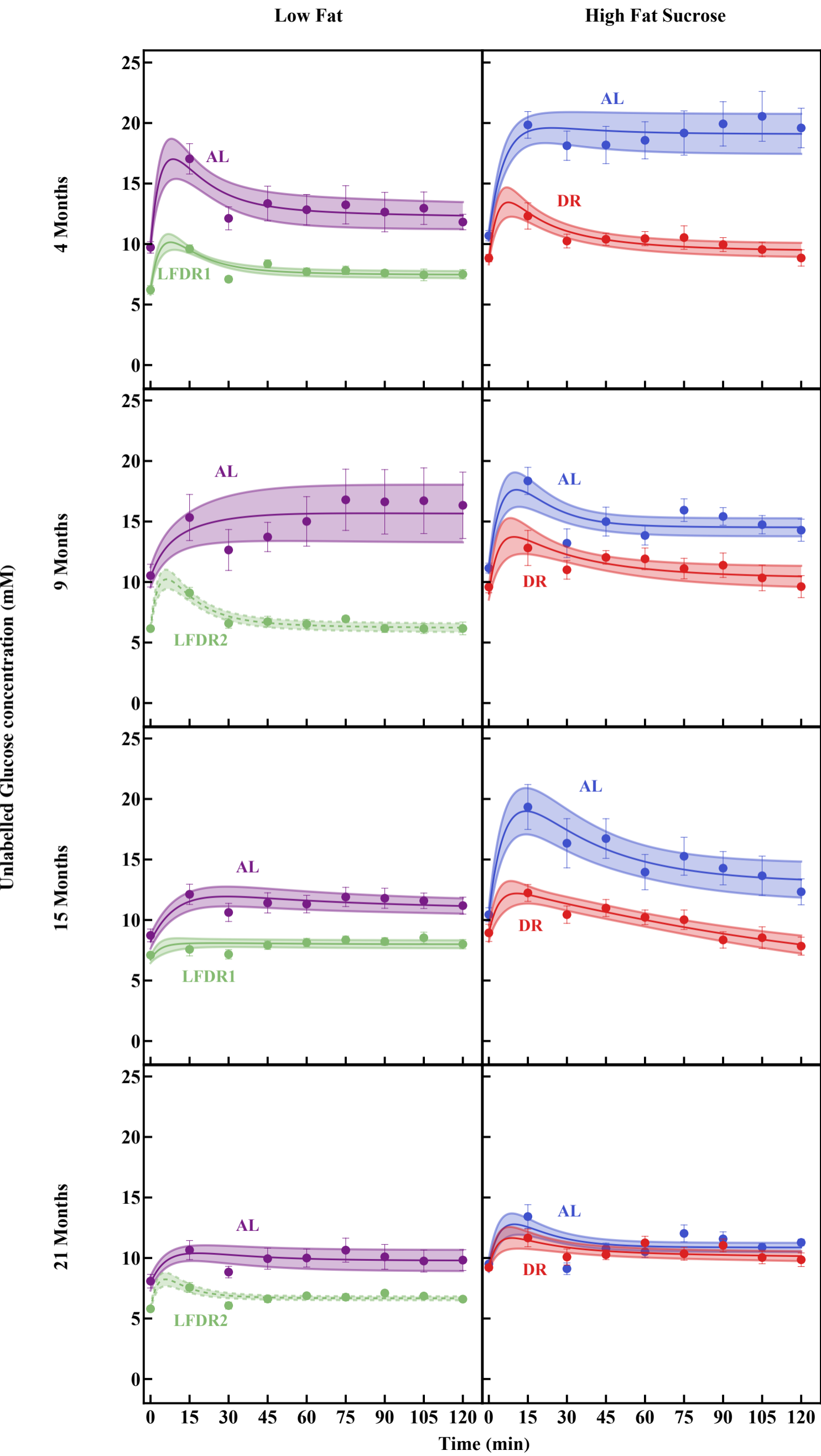

Supplement: Supplementary file 4 — Figure S4: Unlabelled glucose fits for each cohort (age and diet paired). Coloured bands indicate the average ± SEM of the mouse fits. Mean for each timepoint ± SEM are shown as the datapoints and error bars respectively. n per cohort: LFAL (4, 15, 21) months = 8, LFAL 9 months = 7. HFSAL (4, 9, 21) months = 8, HFSAL 15 months = 7. LFDR1 (4, 15) months = 8. LFDR2 9 months = 6, LFDR2 21 months = 8. HFSDR (4, 9, 15, 21) months = 8. [file ACEL-24-e70285-s005.pdf]

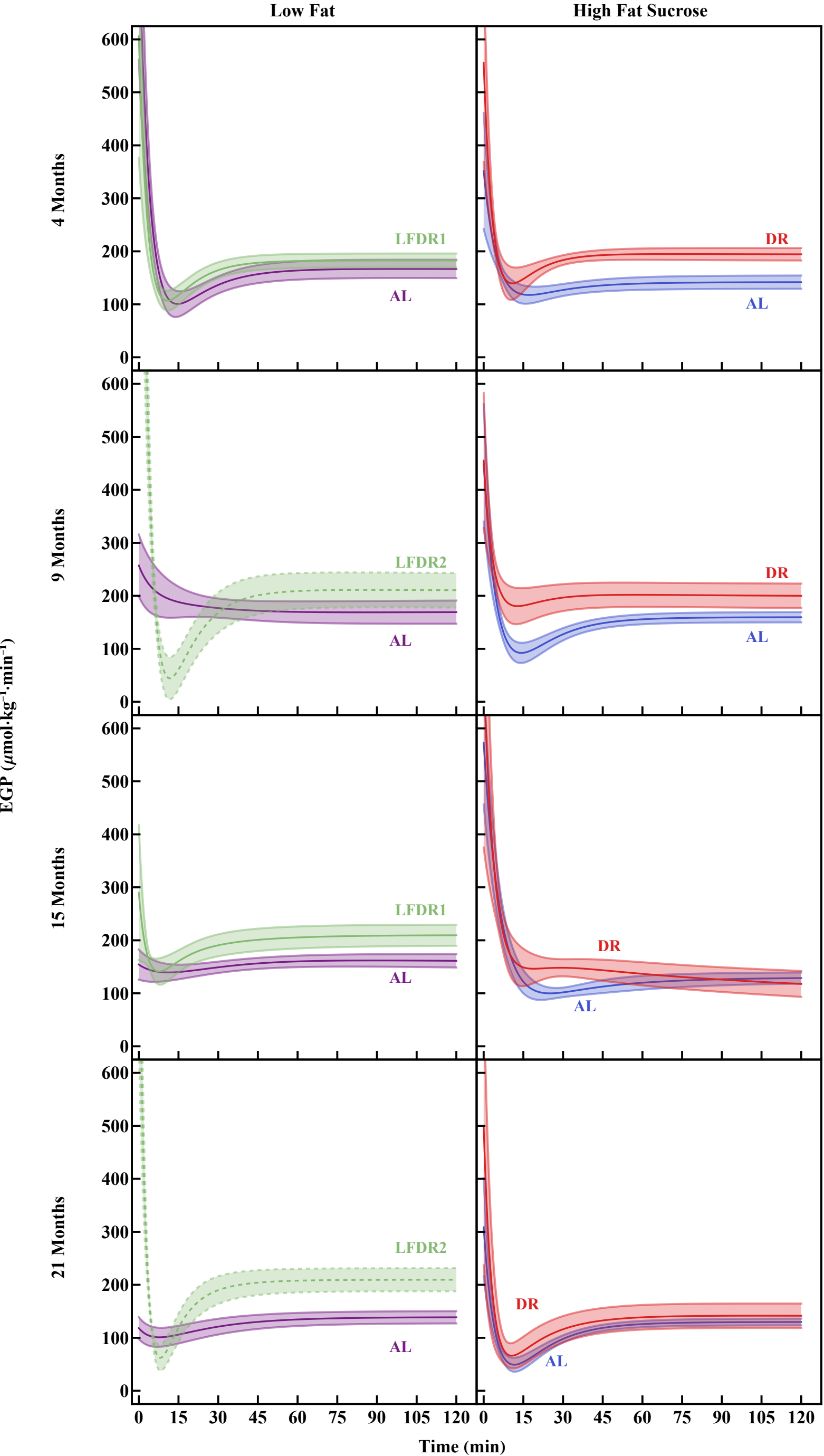

Supplement: Supplementary file 5 — Figure S5: Age and diet paired cohort specific EGP (normalised to BW) time courses. The coloured bands indicate the average ± SEM of the individual mouse EGP time courses in each cohort. n per cohort: EGP: LFAL (4, 15, 21) months = 8, LFAL 9 months = 7. HFSAL (4, 9, 21) months = 8, HFSAL 15 months = 7. LFDR1 (4, 15) months = 8. LFDR2 9 months = 6, LFDR2 21 months = 8. HFSDR (4, 9, 15, 21) months = 8. Significant ART‐ANOVA results for steady‐state specific EGP: pDR = 0.00084144, pDiet = 0.00183543, pDietxAge = 0.03077719. [file ACEL-24-e70285-s011.pdf]

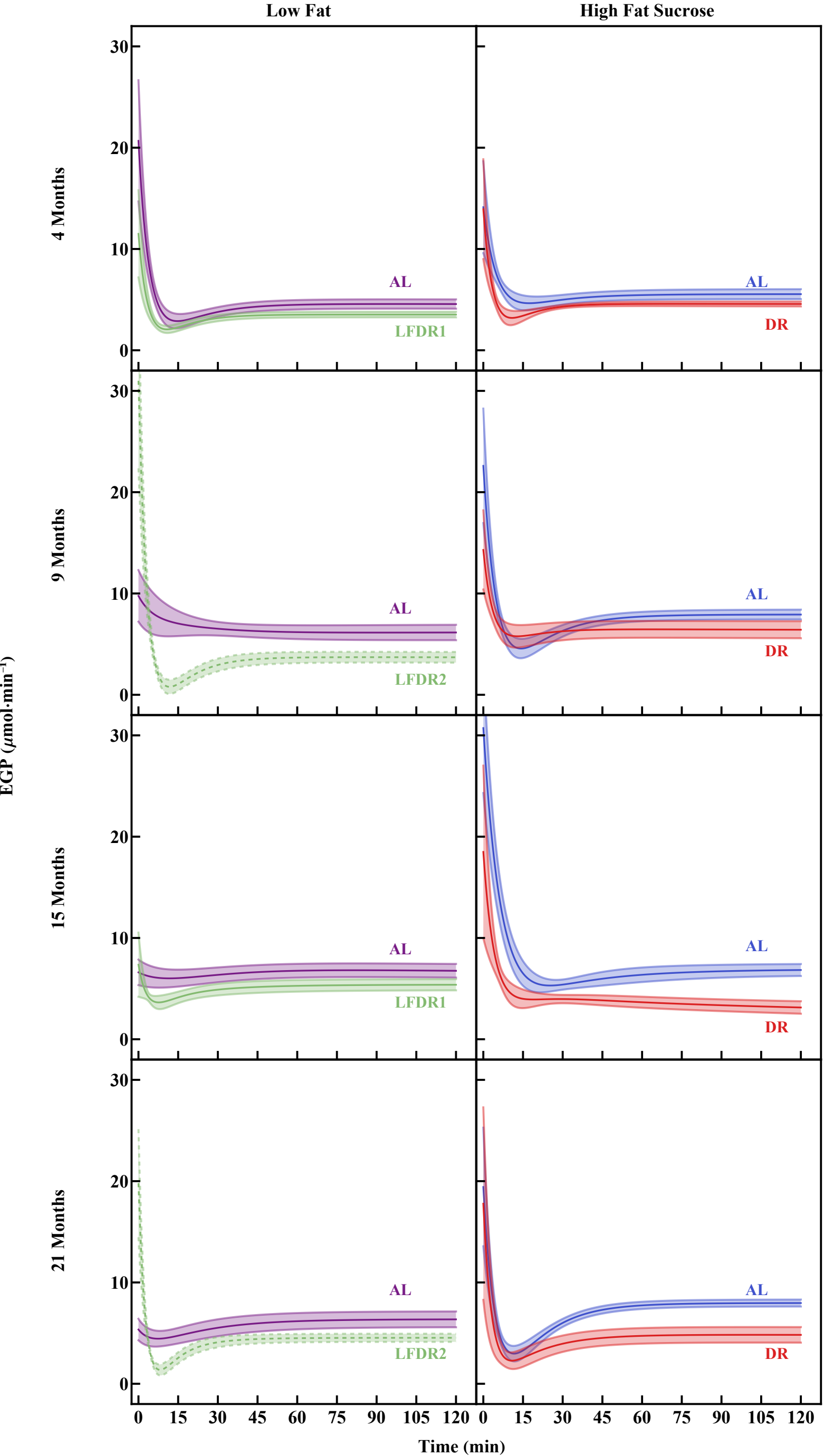

Supplement: Supplementary file 6 — Figure S6: Age and diet paired cohort EGP time courses not normalised to BW. The coloured bands indicate the average ± SEM of the individual mouse EGP time courses in each cohort. LFDR1 cohorts are annotated as DR1 and LFDR2 cohorts annotated as DR2 with the time‐courses shown as dashed lines. n per cohort: LFAL (4, 15, 21) months = 8, LFAL 9 months = 7. HFSAL (4, 9, 21) months = 8, HFSAL 15 months = 7. LFDR1 (4, 15) months = 8. LFDR2 9 months = 6, LFDR2 21 months = 8. HFSDR (4, 9, 15, 21) months = 8. Significant ANOVA results for time‐averaged EGP not normalised to BW: pDR = 7.452 x 10^–9^, pDiet = 0.0104636, pAge = 0.0004068, pDietxAge = 0.0143884. Significant ANOVA results for steady‐state EGP not normalised to BW: pDR = 2.972 x 10^–9^, pDiet = 0.0110855, pAge = 0.0002709, pDietxAge = 0.0017708. [file ACEL-24-e70285-s013.pdf]

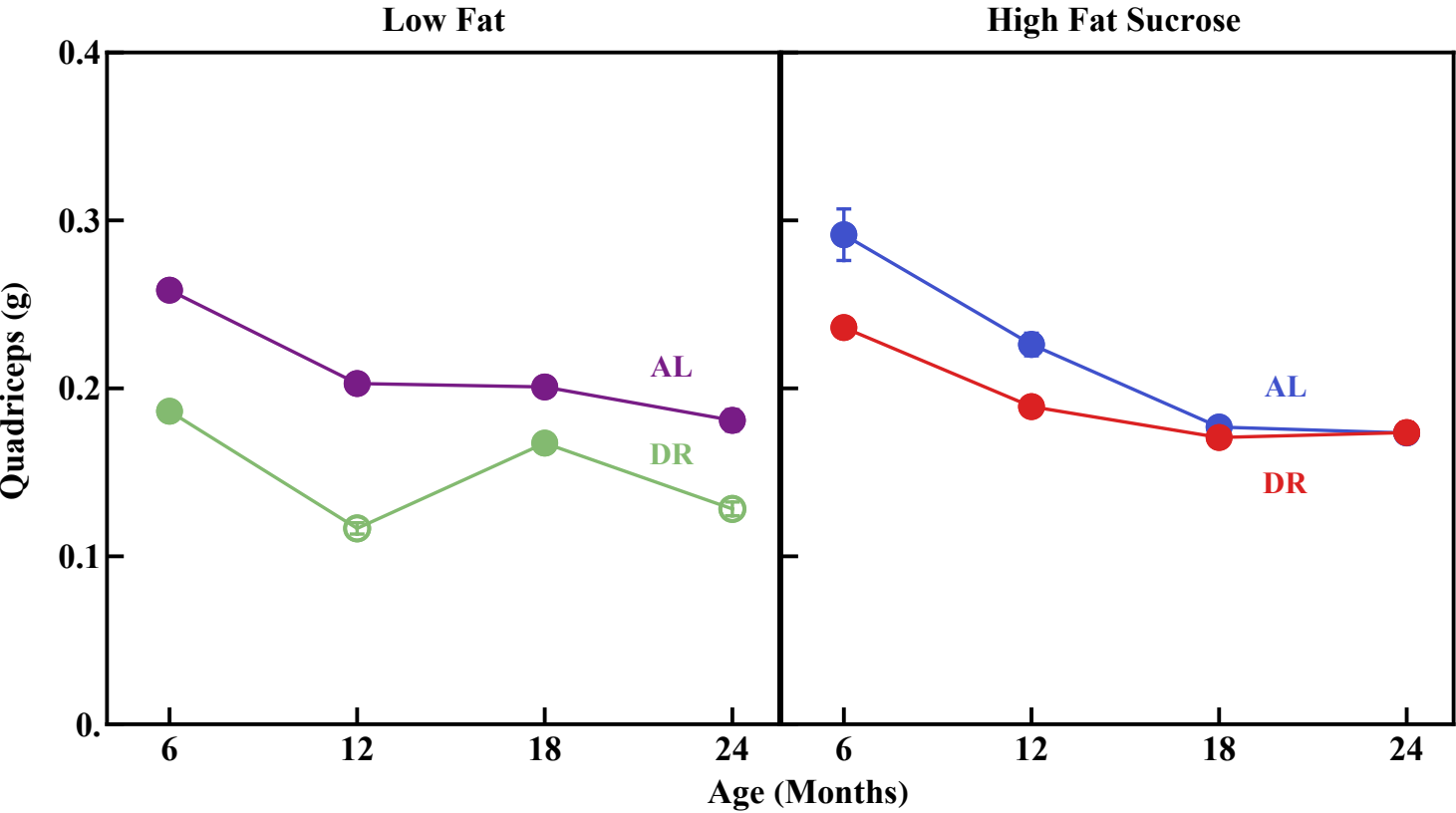

Supplement: Supplementary file 7 — Figure S7: Mouse average quadriceps mass. Mean quadriceps mass (data shown as discs) ± SEM for each cohort. Data shown for 6‐month cohorts are from the quadriceps of 1 leg, not the mean of 2 legs. LFDR2 cohorts (receiving 60% of LFAL calories i.e. more restricted than LFDR1 cohorts) are indicated by the open symbols. n per cohort: LFAL (4, 9, 21) months = 7, LFAL 15 months = 8. HFSAL (4, 9) months = 8, HFSAL (15, 21) months = 7. LFDR1 (4, 15) months = 7, LFDR2 (9, 21) months = 7. HFSDR (4, 21) = 7, HFSDR (9, 15) = 8. [file ACEL-24-e70285-s003.pdf]

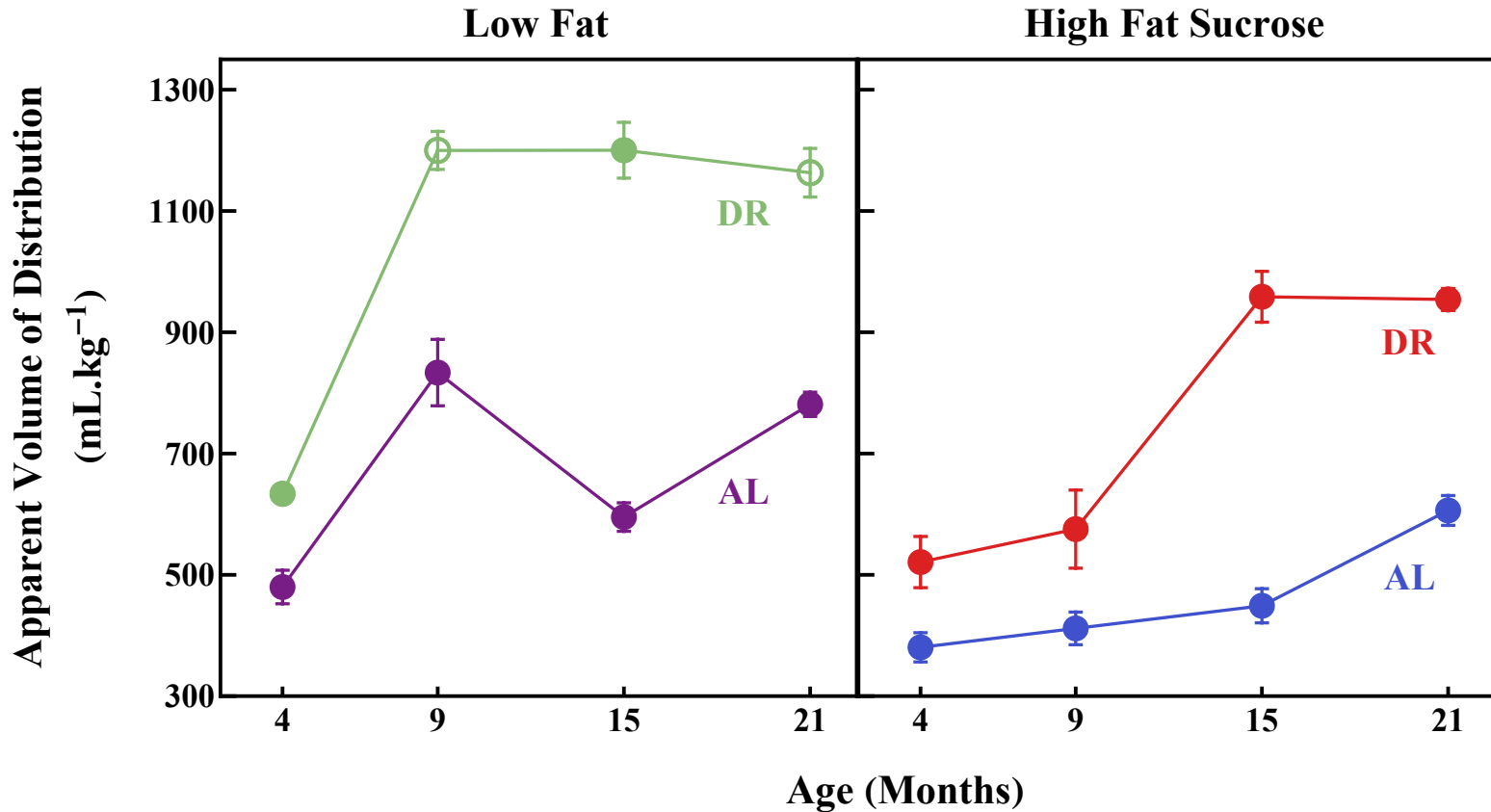

Supplement: Supplementary file 8 — Figure S8: Apparent volume of distribution (normalised to BW). Mean apparent volume of distribution normalised to BW ± SEM. LFDR2 cohorts are indicated by the open symbols. n per cohort: LFAL (4, 15, 21) months = 8, LFAL 9 months = 7. HFSAL (4, 9, 21) months = 8, HFSAL 15 months = 7. LFDR1 (4, 15) months = 8. LFDR2 9 months = 6, LFDR2 21 months = 8. HFSDR (4, 9, 15, 21) months = 8. Significant ANOVA results: pDR < 2.2 x 10^–16^, pDiet < 2.2 x 10^–16^, pAge < 2.2 x 10^–16^, pDRxDiet = 0.01729, pDRxAge = 5.597 x 10^–12^, pDietxAge = 1.5 x 10^–12^. [file ACEL-24-e70285-s014.pdf]

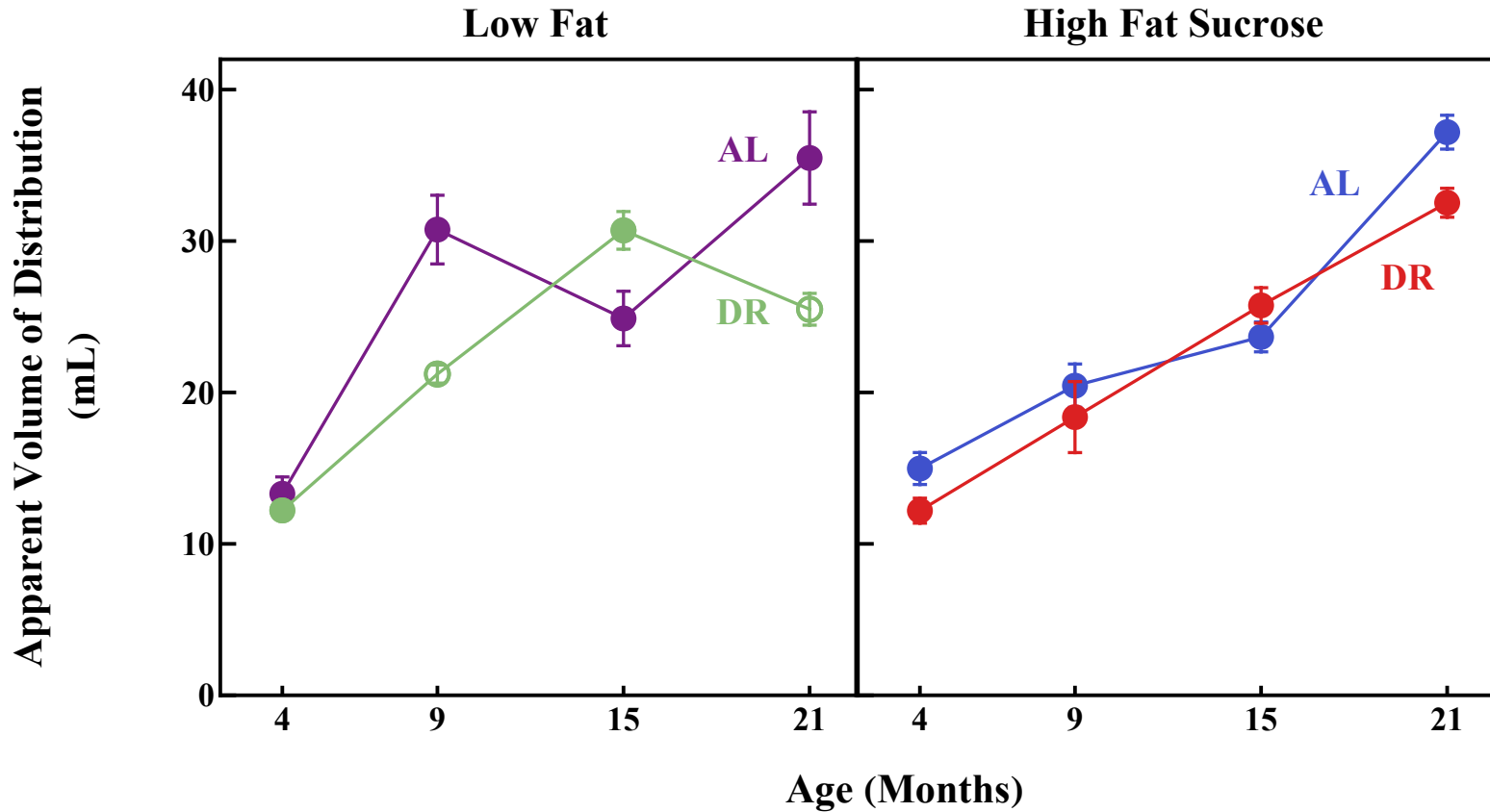

Supplement: Supplementary file 9 — Figure S9: Apparent volume of distribution (not normalised to BW). Mean apparent volume of distribution (not normalised to BW) ± SEM. LFDR2 cohorts are indicated by the open symbols. n per cohort: LFAL (4, 15, 21) months = 8, LFAL 9 months = 7. HFSAL (4, 9, 21) months = 8, HFSAL 15 months = 7. LFDR1 (4, 15) months = 8. LFDR2 9 months = 6, LFDR2 21 months = 8. HFSDR (4, 9, 15, 21) months = 8. Significant ANOVA results: pDR = 0.002064, pAge < 2.2 x 10^–16^, pDRxAge = 2.014 x 10^–6^, pDietxAge = 6.824 x 10^–5^, pDRxDietxAge = 0.028680. [file ACEL-24-e70285-s009.pdf]

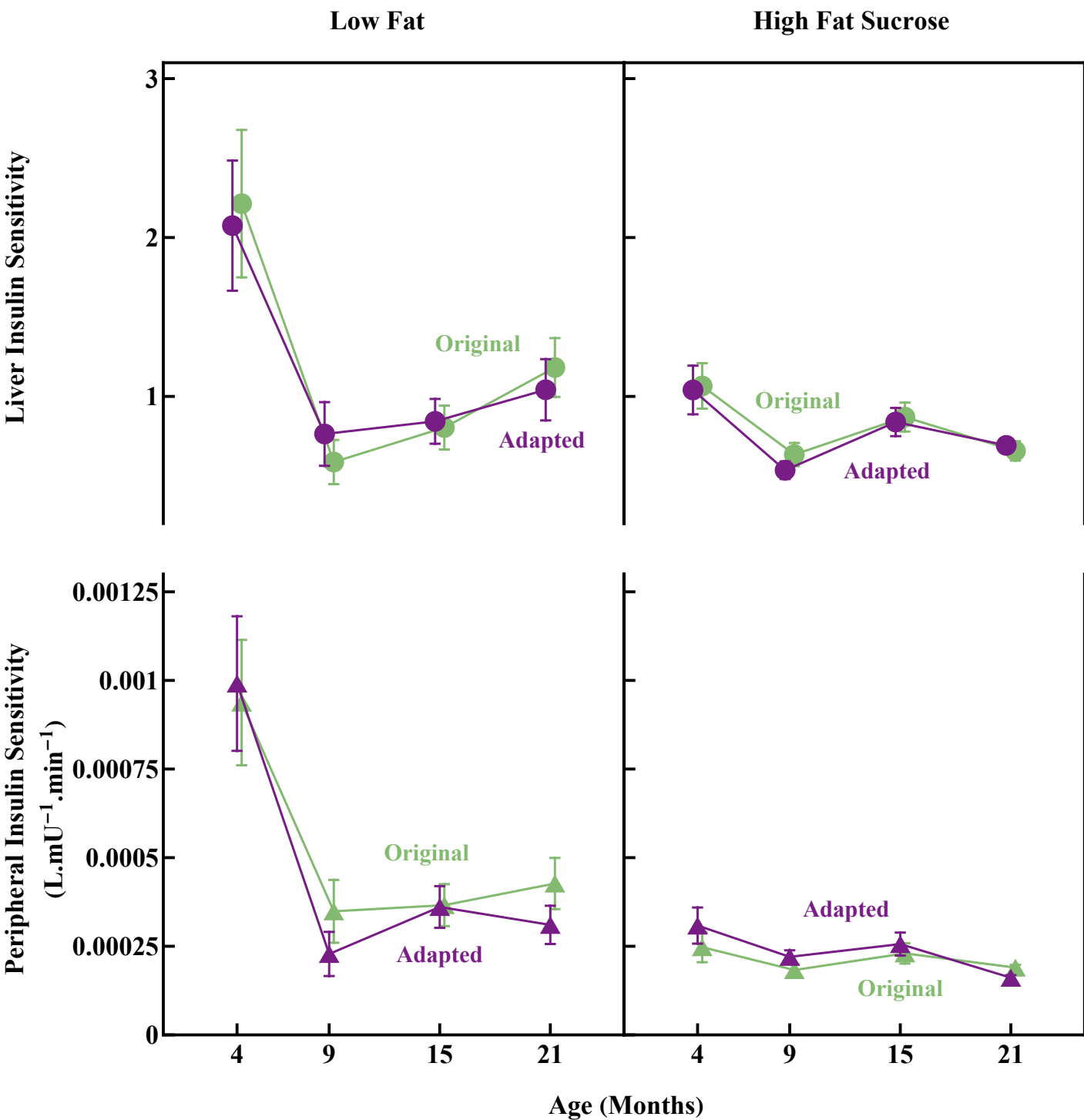

Supplement: Supplementary file 10 — Figure S10: ISL (top panel, data shown as discs) and ISP (bottom panel, data shown as triangles) for the control cohorts analysed by Vieira‐Lara and colleagues (Vieira‐Lara et al. 2023) using their original modelling approach and this adapted approach. ISL is dimensionless as it is normalised to the cohorts of the study. Calculated sample size for each cohort: Original ISL: LFAL 4 months = 9, LFAL (9, 15) months = 7, LFAL 21 months = 8. HFSAL (4, 9) months = 7, HFSAL 15 months = 8, HFSAL 21 months = 6. Adapted ISL: LFAL (4, 15, 21) months = 8, LFAL 9 months = 7. HFSAL (4, 9, 15, 21) months = 8. Original and adapted ISP: LFAL (4, 15, 21) months = 8, LFAL 9 months = 7. HFSAL (4, 15, 21) months = 8, HFSAL 9 months = 7. [file ACEL-24-e70285-s012.pdf]
