# Supplementary material for: Long‐Term Dietary Restriction Has a Strong and Positive Effect on Both Hepatic and Peripheral Insulin Sensitivity, in an Age‐ and Diet‐Dependent Manner
Source: Aging Cell. 2025 Nov 10;24(12):e70285. doi: 10.1111/acel.70285 (PMC12686546; doi:10.1111/acel.70285)
Supplement: Supplementary file 2 — Figure S2: Effect of diet and dietary provision on fitted model parameters for apparent glucose absorption and elimination rate constants. Apparent absorption (ka, top panel, data shown as discs) and elimination (k2, bottom panel, data shown as triangles) rate constants obtained from fitting tracer glucose time course data. Means ± SEM are shown for each cohort. The LFDR cohorts have been separated into 2 subcategories based on the different degree of DR applied. LFDR1: LFDR cohorts receiving 60% of HFSAL calories indicated by the closed symbols at 4‐ and 15‐months. LFDR2: LFDR cohorts receiving 60% of LFAL calories (more restrictive), indicated by the open symbols at 9‐ and 21‐months. n per cohort: LFAL (4, 15, 21) months = 8, LFAL 9 months = 7. HFSAL (4, 9, 21) months = 8, HFSAL 15 months = 7. LFDR1 (4, 15) months = 8. LFDR2 9 months = 6, LFDR2 21 months = 8. HFSDR (4, 9, 15, 21) months = 8. Significant ANOVA results for k2: pDR = 1.304 x 10^–6^, pAge =3.499 x 10^–11^, pDRxAge =3.552 x 10^–7^, pDietxAge = 3.064 x 10^–5^, pDRxDietxAge = 0.004624. [file ACEL-24-e70285-s006.pdf]

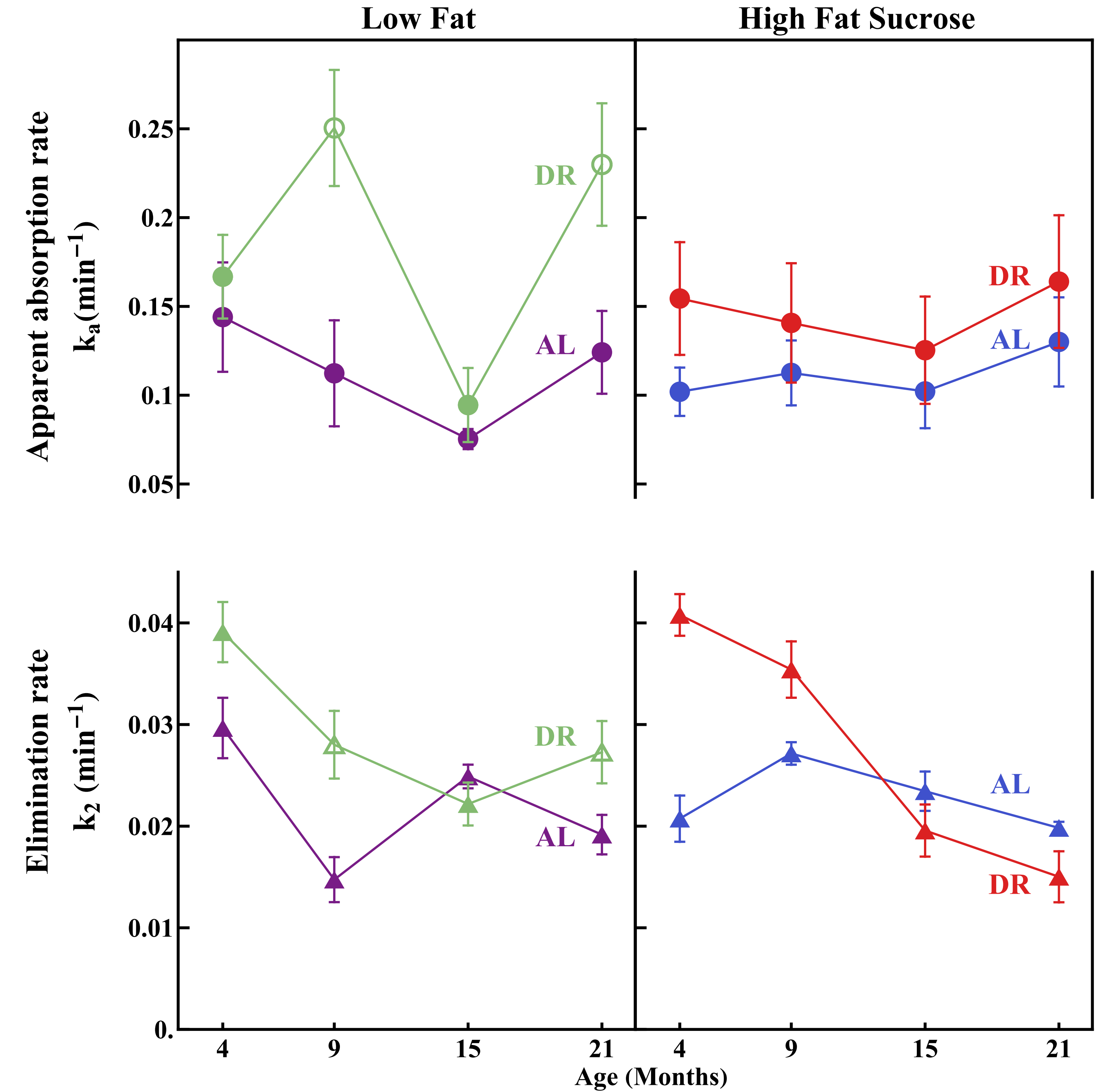
Low Fat
High Fat Sucrose
Apparent absorption rate
$k_a (min^{-1})$
DR
AL
DR
AL
0.25
0.2
0.15
0.1
0.05
Elimination rate
k_2 (min^{-1})
DR
AL
AL
DR
0.04
0.03
0.02
0.01
0.
4
9
15
21
4
9
15
21
Age (Months)
