# Supplementary material for: Long‐Term Dietary Restriction Has a Strong and Positive Effect on Both Hepatic and Peripheral Insulin Sensitivity, in an Age‐ and Diet‐Dependent Manner
Source: Aging Cell. 2025 Nov 10;24(12):e70285. doi: 10.1111/acel.70285 (PMC12686546; doi:10.1111/acel.70285)
Supplement: Supplementary file 11 — Table S1: Parameter k2 ANOVA results. Table S2: Peak tracer glucose concentration ANOVA results. Table S3: Average insulin concentration ANOVA results. Table S4: Normalised apparent distribution volume ANOVA results. Table S5: Apparent distribution volume (not normalised to BW) ANOVA results. Table S6: Average specific EGP ANOVA results. Table S7: Average specific EGP ART‐ANOVA results. Table S8: Average EGP (not normalised to BW) ANOVA results. Table S9: Steady‐state specific EGP ART‐ANOVA results. Table S10: Steady‐state EGP (not normalised to BW) ANOVA results. Table S11: ISP ANOVA results. Table S12: ISL ANOVA results. Table S13: Identifiability of ka and k2 parameters (with 95% confidence) expressed as a proportion of mice in each cohort. Table S14: Sample size for each cohort for each metric/measurement. [file ACEL-24-e70285-s016.docx]

Supplementary Materials

Mice and Experimental Breakdown

Male C57BL6/JOlaHsd mice were housed individually from weaning onwards and divided into four main cohorts according to either an LF or HFS diet with added sucrose (LF, 6% fat, AMII 2141, 17.5 kJ/gram and HFS, 45% fat, 4031.09, 19.1 kJ/gram each from HopeFarms BV, Woerden, NL) with or without DR as previously described by Reijne and colleagues (Reijne et al., 2022). The 45% fat content is considered to be most relevant for comparison to human physiology (Speakman, 2019). The DR cohorts received their food between circadian time 9 and 10, with 12 the beginning of the dark phase (Reijne et al., 2022). The HFS restricted cohorts (HFSDR) received 60% of the calories consumed by the HFSAL cohorts, while the LFDR cohorts received either 60% of the calories from the HFSAL cohorts (equivalent to approximately 70% of the calories consumed by the LFAL cohorts), or 60% of the calories from the LFAL cohorts, LFDR1 and LFDR2 respectively. The calories consumed by the AL cohorts were measured every 1.5-2 weeks initially, then every 3-4 weeks. Daily food intake was calculated and used to determine the amount to be provided to the DR cohorts. A total of 125 mice were included in this study, with 62 in DR and 63 in AL cohorts. These cohorts were then further subdivided according to the age at which the OGTT was performed.

Modelling of Labelled and Unlabelled Glucose data

The modelling approach is based on (Vieira-Lara et al., 2023) and considers two compartments explicitly, the gastrointestinal (GI) tract and blood plasma compartments (Figure 1A), with transfer of glucose from the GI tract to the blood plasma described by the absorption rate, with rate constant k_1_. Loss of glucose from the GI tract prior to detection in the plasma is accounted for with a loss rate constant (k_L_), leading to a total apparent glucose absorption rate from the GI tract dependent on the rate constant k_a_ = k_1_ + k_L_. The elimination of glucose from the blood plasma is dependent on the elimination rate constant k_2_. The OGTT glucose time course data was fitted to obtain k_a_ and k_2_. The distribution volume, assuming a fixed bioavailability of the oral glucose of 80%, as well as the EGP and IS indices were then calculated (Vieira-Lara et al., 2023). A detailed description of the refined fitting procedure is available in the Supplementary Materials including a comparison to the original approach (Vieira-Lara et al., 2023).

Data Analysis

Model fits and data visualization were performed using Wolfram Mathematica® and the functions NonLinearModelFit and NMinimize. 3-way (ART-)ANOVAs (type three) were performed in R (4.3.2) for k_2_, peak tracer glucose, average insulin concentrations, calculated apparent distribution volumes, and average EGP. A significant effect is considered here to be one with p < 0.05. Significant results are included in the main text and figure captions. Due to limited identifiability of k_a_ (discussed in more detail in subsequent sections) statistical analysis of this parameter was precluded.

Analysis of k_2_

3-way ANOVA of the fitted tracer k_2_ parameters was performed with both normality (rstatix::shapiro_test and stats::shapiro.test, p = 0.1335) and homogeneity of variance (rstatix::levene_test, p = 0.291) assumptions upheld.

Table S1. Parameter k_2_ ANOVA results.

|  | Sum Sq | Df | F value | p |
| --- | --- | --- | --- | --- |
| DR | 0.001097 | 1 | 26.2828 | 1.304 x10^-6^ |
| Diet | 0.000005 | 1 | 0.1094 | 0.741516 |
| Age | 0.002751 | 3 | 21.9716 | 3.499 x10^-11^ |
| DRxDiet | 0.000035 | 1 | 0.8316 | 0.363840 |
| DRxAge | 0.001591 | 3 | 12.7083 | 3.552 x10^-7^ |
| DietxAge | 0.001094 | 3 | 8.7379 | 3.064 x10^-5^ |
| DRxDietxAge | 0.000574 | 3 | 4.5849 | 0.004624 |
| Residuals | 0.004507 | 108 |  |  |

Analysis of peak tracer glucose

3-way ANOVA was performed, however, normality and homogeneity of variance assumptions were not upheld (p = 0.008043 and p = 0.00000104, respectively). Two extreme outliers were removed (determined using rstatix:: identify_outliers) and the ANOVA reperformed with the same components determined to be significant, but normality and homogeneity of variance assumptions were still not upheld (p = 0.003361 and p = 0.000000304, respectively). ART-ANOVA (from ARTool, a non-parametric alternative to ANOVA) was then applied to the data both with and without the extreme outliers with both results indicating the same significant components as were identified by the ANOVAs. This confirms the results are robust and subsequently the ANOVA results are reported.

Table S2. Peak tracer glucose concentration ANOVA results

|  | Sum Sq | Df | F value | p |
| --- | --- | --- | --- | --- |
| DR | 9.207 | 1 | 53.7211 | 4.440 x10^-11^ |
| Diet | 6.842 | 1 | 39.9203 | 6.052 x10^-9^ |
| Age | 7.360 | 3 | 14.3143 | 6.416 x10^-8^ |
| DRxDiet | 0.402 | 1 | 2.3430 | 0.1288 |
| DRxAge | 0.663 | 3 | 1.2896 | 0.2817 |
| DietxAge | 0.993 | 3 | 1.9310 | 0.1289 |
| DRxDietxAge | 0.045 | 3 | 0.0880 | 0.9665 |
| Residuals | 18.510 | 108 |  |  |

Analysis of average insulin concentrations

When performing ANOVA on the average plasma insulin concentrations, IS_P_, and IS_L_, the 9-month cohorts were excluded due to the lack of insulin data for the 9-month LFDR cohort. ANOVA was performed on the average insulin concentrations, however, homogeneity of variance and normality assumptions were not upheld (p = 0.00443 and p = 0.000137, respectively). An ART-ANOVA was performed on the data with the same components determined to be significant as were identified by the ANOVA. For this reason, the results of the ANOVA are reported.

Table S3. Average insulin concentration ANOVA results

|  | Sum Sq | Df | F value | p |
| --- | --- | --- | --- | --- |
| DR | 79190 | 1 | 264.5404 | < 2.2 x10^-16^ |
| Diet | 12963 | 1 | 43.3033 | 4.483 x10^-9^ |
| Age | 9906 | 2 | 16.5460 | 9.690 x10^-7^ |
| DRxDiet | 5347 | 1 | 17.8620 | 6.255 x10^-5^ |
| DRxAge | 6381 | 2 | 10.6576 | 7.882 x10^-5^ |
| DietxAge | 2054 | 2 | 3.4314 | 0.03717 |
| DRxDietxAge | 951 | 2 | 1.5890 | 0.21050 |
| Residuals |  | 80 |  |  |

Analysis of apparent distribution volume

Apparent distribution volume (normalised to BW) was analysed with a 3-way ANOVA. All factors excluding the three-way interaction were determined to be significant, however, the normality assumption was not upheld (p = 0.00216). ART-ANOVA was then applied with the same components determined to be significant as identified by the ANOVA, confirming the robustness of the results. The results of the ANOVA are subsequently reported.

Table S4. Normalised apparent distribution volume ANOVA results

|  | Sum Sq | Df | F value | p |
| --- | --- | --- | --- | --- |
| DR | 3427733 | 1 | 349.6560 | < 2.2 x10^-16^ |
| Diet | 1984354 | 1 | 202.4199 | < 2.2 x10^-16^ |
| Age | 2492904 | 3 | 84.7654 | < 2.2 x10^-16^ |
| DRxDiet | 57308 | 1 | 5.8459 | 0.01729 |
| DRxAge | 705878 | 3 | 24.0017 | 5.597 x10^-12^ |
| DietxAge | 750010 | 3 | 25.5023 | 1.500 x10^-12^ |
| DRxDietxAge | 40434 | 3 | 1.3749 | 0.25438 |
| Residuals | 1058741 | 108 |  |  |

Apparent distribution volume not normalised to BW was analysed using ANOVA, however both the assumption of normality and homogeneity of variance were not upheld (p = 0.000843 and p = 0.000958, respectively). An extreme outlier was removed and the analysis reperformed. The three-way interaction was no longer significant (the other components identified as significant with the outlier included remained significant), however, normality and homogeneity of variance were still not upheld (p = 0.00450 and p = 0.0000778, respectively). ART-ANOVA was then applied to the unfiltered data (and again on the data with the extreme outlier excluded) with the same significant components identified as determined by the ANOVA on the full dataset, confirming the robustness of the results. Subsequently, the results of the ANOVA on the unfiltered data are reported.

Table S5. Apparent distribution volume (not normalised to BW) ANOVA results

|  | Sum Sq | Df | F value | p |
| --- | --- | --- | --- | --- |
| DR | 238 | 1 | 13.4596 | 0.0003803 |
| Diet | 38 | 1 | 2.1712 | 0.1435247 |
| Age | 6353 | 3 | 119.5986 | < 2.2 x10^-16^ |
| DRxDiet | 26 | 1 | 1.4779 | 0.2267546 |
| DRxAge | 586 | 3 | 11.0368 | 2.228 x10^-6^ |
| DietxAge | 513 | 3 | 9.6585 | 1.058 x10^-5^ |
| DRxDietxAge | 166 | 3 | 3.1200 | 0.0290574 |
| Residuals | 1912 | 108 |  |  |

Summary:

DR cohorts had elevated apparent volume of distribution of the tracer, when normalised to BW (Figure S8), compared to AL cohorts (p_DR_ < 2.2 x 10^-16^), with this modulated by Diet and Age (p_DRxDiet_ = 0.01729, p_DRxAge_ = 5.597 x 10^-12^). An LF diet elevated the normalised apparent volume of distribution compared to an HFS diet, with age modulating this effect (p_Diet_ < 2.2 x 10^-16^, p_DietxAge_ = 1.5 x 10^-12^). Age also had a significant effect on distribution volume (p_Age_ < 2.2 x 10^-16^), with older cohorts having generally elevated volumes compared to younger cohorts. The apparent volume of distribution not normalised to BW (Figure S9) differed between DR and AL cohorts in an age dependent manner, with diet further modulating this effect (p_DR_ = 0.0003803, p_DRxAge_ = 2.228 x10^-6^, p_DRxDietxAge_ = 0.0290574). This effect is notably reduced compared to that of the normalised volumes of distribution. Age had a significant effect (p_Age_ < 2.2 x 10^-16^), with older cohorts having generally elevated volumes compared to younger cohorts but this effect was modulated by diet (p_DietxAge_ = 1.058 x10^-5^).

Analysis of average EGP

The average specific EGP was analysed with an ANOVA, with the normality assumption not upheld (p = 0.0158). The analysis was repeated after removing 2 extreme outliers with the normality assumption now upheld (p = 0.0644) but the homogeneity of variance assumption no longer satisfied (p = 0.0135). ART-ANOVA was then applied to the average specific EGP data with almost the same significant components identified compared to the ANOVA, except for the effect of Age. Age was determined to be significant using the ANOVA (p = 0.0283487) but not when using ART-ANOVA (p = 0.06054375). The results of the ART-ANOVA are reported in the main text, however, the significance of age according to ANOVA as well as lack of significance according to ART-ANOVA is indicated in the caption of Figure 2. Evidence for the effect of ageing on average specific EGP is not as robust and depends on the assumptions used in the statistical analysis. Both the ANOVA and ART-ANOVA results are shown in Tables S6 and S7, respectively.

Table S6. Average specific EGP ANOVA results

|  | Sum Sq | Df | F value | p |
| --- | --- | --- | --- | --- |
| DR | 32781 | 1 | 14.9921 | 0.0001853 |
| Diet | 16690 | 1 | 7.6330 | 0.0067383 |
| Age | 20595 | 3 | 3.1397 | 0.0283487 |
| DRxDiet | 3 | 1 | 0.0015 | 0.9690529 |
| DRxAge | 417 | 3 | 0.0636 | 0.9789607 |
| DietxAge | 11333 | 3 | 1.7276 | 0.1656357 |
| DRxDietxAge | 11249 | 3 | 1.7148 | 0.1682611 |
| Residuals | 236150 | 108 |  |  |

Table S7. Average specific EGP ART-ANOVA results

|  | Df | F value | p |
| --- | --- | --- | --- |
| DR | 1 | 12.68934 | 0.00054863 |
| Diet | 1 | 6.88167 | 0.00996772 |
| Age | 3 | 2.53634 | 0.06054375 |
| DRxDiet | 1 | 0.28204 | 0.59645910 |
| DRxAge | 3 | 0.24094 | 0.86760612 |
| DietxAge | 3 | 2.30988 | 0.08040645 |
| DRxDietxAge | 3 | 1.89327 | 0.13504037 |
| Residuals | 108 |  |  |

The average EGP not normalised to BW was analysed with ANOVA, with both normality and homogeneity of variance assumptions upheld (p = 0.488 and p = 0.138, respectively). Subsequently the ANOVA results are reported.

Table S8. Average EGP (not normalised to BW) ANOVA results

|  | Sum Sq | Df | F value | p |
| --- | --- | --- | --- | --- |
| DR | 104.9 | 1 | 44.2764 | 1.219 x10^-9^ |
| Diet | 17.6 | 1 | 7.4372 | 0.007458 |
| Age | 38.0 | 3 | 5.3535 | 0.001782 |
| DRxDiet | 0.0 | 1 | 0.0014 | 0.970654 |
| DRxAge | 8.3 | 3 | 1.1637 | 0.327097 |
| DietxAge | 26.7 | 3 | 3.7633 | 0.012934 |
| DRxDietxAge | 14.2 | 3 | 2.0023 | 0.117977 |
| Residuals | 255.8 | 108 |  |  |

Analysis of steady-state EGP

The steady-state (last 30 minutes) specific EGP was analysed with ANOVA, with the normality assumption not upheld (p = 0.00521). Three extreme outliers were removed and the data reanalysed, however both normality and homogeneity of variance assumptions were no longer upheld (p = 0.00994 and p = 0.0409, respectively). ART-ANOVA was applied to the data with and without the extreme outliers with identical significant components determined. Consequently, the ART-ANOVA results on the full dataset are reported.

Table S9. Steady-state specific EGP ART-ANOVA results

|  | Df | F value | p |
| --- | --- | --- | --- |
| DR | 1 | 11.8000 | 0.00084144 |
| Diet | 1 | 10.2058 | 0.00183543 |
| Age | 3 | 2.3550 | 0.07599101 |
| DRxDiet | 1 | 2.2577 | 0.13586874 |
| DRxAge | 3 | 0.4572 | 0.71276341 |
| DietxAge | 3 | 3.0744 | 0.03077719 |
| DRxDietxAge | 3 | 2.2896 | 0.08247178 |
| Residuals | 108 |  |  |

Steady-state EGP values not normalised to BW were analysed with ANOVA and both normality and homogeneity of variance assumptions were upheld (p = 0.458 and p = 0.134, respectively). Subsequently, the ANOVA results are reported.

Table S10. Steady-state EGP (not normalised to BW) ANOVA results

|  | Sum Sq | Df | F value | p |
| --- | --- | --- | --- | --- |
| DR | 120.4 | 1 | 46.6405 | 5.215 x10^-10^ |
| Diet | 19.0 | 1 | 7.3715 | 0.007717 |
| Age | 43.0 | 3 | 5.5480 | 0.001402 |
| DRxDiet | 2.8 | 1 | 1.0770 | 0.301685 |
| DRxAge | 11.5 | 3 | 1.4845 | 0.222867 |
| DietxAge | 42.3 | 3 | 5.4623 | 0.001558 |
| DRxDietxAge | 10.8 | 3 | 1.3971 | 0.247677 |
| Residuals | 278.8 | 108 |  |  |

Summary:

The specific EGP rates at steady-state, i.e. at the end of the time course, (analysed with ART-ANOVA) were elevated for DR compared to AL (p_DR_ = 0.00084144, Figure S5), and LF compared to HFS in an age-dependent manner (p_Diet_ = 0.00183543, p_DietxAge_ = 0.030777). In contrast, steady-state EGP not normalised to BW (analysed with ANOVA) showed a small reduction by DR compared to AL (p_DR_ = 5.215 x10^-10^ ) (Figure S6), with HFS cohorts generally slightly elevated compared to LF cohorts (p_Diet_ = 0.007717), although this was modulated by age (p_DietxAge_ = 0. 001558). Steady-state EGP also varied with age (p_Age_ = 0. 001402).

Peripheral and liver insulin sensitivity indices (IS_P_ and IS_L_ respectively) were calculated in Wolfram Mathematica® using the product or ratio of the means and distributions (Vieira-Lara et al., 2023) and a 3-way ANOVA of the summary statistics for each cohort was performed in GraphPad Prism (10.4.1) (Vieira-Lara et al., 2023). The results are shown in Tables S11 and S12. The sample size for glucose measurements, k_a_, k_2_, EGP, apparent volume of distribution, quadricep, insulin, IS_P_, and IS_L_ are shown in Table S14.

Table S11. IS_P_ ANOVA results

|  | Sum Sq | Df | F value | p |
| --- | --- | --- | --- | --- |
| DR | 6.946 x 10^-5^ | 1 | 127.8 | < 0.0001 |
| Diet | 1.178 x10^-5^ | 1 | 21.68 | < 0.0001 |
| Age | 1.448 x10^-5^ | 2 | 13.32 | < 0.0001 |
| DRxDiet | 3.830 x10^-6^ | 1 | 7.049 | 0.0095 |
| DRxAge | 4.466 x10^-6^ | 2 | 4.110 | 0.0199 |
| DietxAge | 2.422 x10^-6^ | 2 | 2.228 | 0.1143 |
| DRxDietxAge | 7.880 x10^-6^ | 2 | 7.252 | 0.0013 |
| Residuals | 4.401 x10^-5^ | 81 |  |  |

Table S12. IS_L_ ANOVA results

|  | Sum Sq | Df | F value | p |
| --- | --- | --- | --- | --- |
| DR | 148.9 | 1 | 87.51 | < 0.0001 |
| Diet | 14.34 | 1 | 8.431 | 0.0047 |
| Age | 6.895 | 2 | 2.027 | 0.1383 |
| DRxDiet | 3.168 | 1 | 1.863 | 0.1761 |
| DRxAge | 10.75 | 2 | 3.159 | 0.0477 |
| DietxAge | 0.7767 | 2 | 0.2283 | 0.7964 |
| DRxDietxAge | 5.900 | 2 | 1.734 | 0.1830 |
| Residuals | 139.5 | 82 |  |  |

Comment on elevated average specific EGP and elevated IS

At first sight, increased EGP and improved insulin sensitivity seems to be counterintuitive. However, it is conceivable that basal (fasting) EGP might be relatively elevated in DR animals due to heightened gluconeogenic drive (e.g., due to a high glucagon to insulin ratio) in extended fasted states. A 2015 mouse study investigated the effect of DR in conjunction with an HF diet and found heightened mRNA expression levels of enzymes involved in hepatic gluconeogenesis in the DR cohort compared to AL cohort (<https://pubmed.ncbi.nlm.nih.gov/25405871/>), thereby substantiating this possibility. Improved insulin sensitivity in DR usually means improved insulin signalling when insulin is present; e.g., during a clamp or a post-meal (OGTT) state. The overall net basal EGP would thus reflect a balance of elevated hormonal (glucagon/insulin) drive and intrinsic hepatic insulin responsiveness during nutrient pulses.

The pool sizes and plasma concentrations were modelled with the following ordinary differential equations (ODEs), assuming a single absorption phase into and elimination phase out of the blood plasma (Vieira-Lara et al., 2023)

$$\frac{d\text{Glc}_{\boldsymbol{Lab, Pool}}\boldsymbol{(}t\boldsymbol{)}}{dt}=-\left( k_{1}+k_{L} \right)\cdot\text{Glc}_{\boldsymbol{Lab,Pool}}\boldsymbol{(}t\boldsymbol{)}$$

$$\frac{d\text{Glc}_{\boldsymbol{Unlab, Pool}}\boldsymbol{(}t\boldsymbol{)}}{dt}=-\left( k_{1}+k_{L} \right)\cdot\text{Glc}_{\boldsymbol{Unlab,Pool}}\boldsymbol{(}t\boldsymbol{)}$$

$$\frac{d\text{Glc}_{\boldsymbol{Lab}}\boldsymbol{(}t\boldsymbol{)}}{dt}=\frac{k_{1}\cdot\text{Glc}_{\boldsymbol{Lab,Pool}}(t)}{Vol}-k_{2}\cdot\text{Glc}_{\boldsymbol{Lab}}(t)$$

$$\frac{d\text{Glc}_{\boldsymbol{Unlab}}\boldsymbol{(}t\boldsymbol{)}}{dt}=\frac{k_{1}\cdot\text{Glc}_{\boldsymbol{Unlab,Pool}}\boldsymbol{(}t\boldsymbol{)}}{Vol}-k_{2}\cdot\text{Glc}_{\boldsymbol{Unlab}}(t)+\text{EGP}\left( t \right)$$

with subscript Pool indicating the amount of the relevant glucose present in the GI Tract in µmol.kg^-1^, Glc_Lab_ the labelled glucose plasma concentration, Glc_Unlab_ the unlabelled glucose plasma concentration, Vol the apparent distribution volume of the plasma compartment (mL.kg^-1^), k_1_ the absorption rate constant, and k_2_ the elimination rate constant. The amount of glucose administered to each mouse was informed by their respective body weights, with the body weights of all mice included in the OGTT shown in Figure S1. Using k_a_ = k_1_ + k_L_, the apparent absorption rate constant, analytical solutions for the plasma glucose concentrations can be expressed as

$$\mathbf{Gl}\mathbf{c}_{\boldsymbol{Lab}}\left( t \right)=C\cdot\left( -e^{-k_{a}t} + e^{-k_{2}t} \right)$$

$$\mathbf{Gl}\mathbf{c}_{\boldsymbol{Unlab}}(t)=C_{0}-C_{1}\cdot e^{-k_{a}t}+C_{2}\cdot e^{-k_{2}t}$$

$$C=-\frac{\text{Glc}_{\boldsymbol{Lab,Pool}}\left( 0 \right)}{Vol}\cdot\frac{k_{a}\cdot F}{k_{2}-k_{a}}$$

$$F=\frac{k_{1}}{k_{1}+k_{L}}$$

with C, C_0_, C_1_, C_2_, k_a_, and k_2_ parameters fitted using available glucose data and F the bioavailability, assumed to be 80% (Vieira-Lara et al., 2023). More information regarding model derivation and validation are provided in a previous publication (Vieira-Lara et al., 2023). In this way, labelled glucose is expected to increase from an initial concentration of 0 mM to a peak, followed by a decline to 0 mM. Similar dynamics were expected for the unlabelled glucose, however, the initial and final concentrations are expected to be non-zero and variable due to basal plasma glucose concentrations and EGP varying between mice.

The tracer data was processed by removing data values corresponding to a percentage enrichment greater than 20% as this is considered erroneous due to the initial bolus tracer concentration being 30% and the volume of the bolus administered being considerably less than the plasma of each mouse. In total, 5 tracer data points across all mice were removed in this manner. The corresponding unlabelled glucose concentrations were then also removed as they are dependent on the tracer concentrations. One mouse was excluded due to a lack of data during the interval where peak glucose concentrations are expected in the blood as this would inhibit accurate identification of parameters during model fitting.

The fits were performed in Wolfram Mathematica^®^ using NonLinearModelFit with method set to “NMinimize” for tracer fits, and NMinimize to reduce a custom objective function for the unlabelled fits. Identifiability analysis was then performed for the tracer parameters according to the Profile Likelihood approach using the sum of squared residuals to calculate the log-likelihood test statistic and subsequent confidence intervals, at a cohort level for the tracer glucose fits, with the range tested for each parameter as follows:

$$0.1\leq C\leq15 0.01\leq k_{a}\leq1 0.001\leq k_{2}\leq1$$

Limited identifiability was observed, consequently, the parameter C was fixed for each cohort such that the “goodness of fit” (as quantified by the sum of squared residuals) was optimised while ensuring the correlation between k_a_ and k_2_ remained lower than 50% (as determined using a Spearman correlation coefficient of 0.5 as the threshold). This was achieved by first performing a parameter scan for a range of C values for each cohort using the mean of the cohort data for each timepoint. The Spearman correlation coefficient was then calculated using 20 000 fits of synthetic data sampled from a normal distribution using the means of the cohort data for each timepoint as well as the standard deviations, assuming a fixed C value. This was performed for several C values, such that a C value could be selected that was as close as possible to the C value providing the best fit to the mean of the cohort data, while also maintaining a Spearman r < 0.5 for the synthetic data fits. Synthetic data was used for this correlation step as it assists with cohorts exhibiting a high correlation between parameters k_a_ and k_2_. Additionally, the relatively small standard deviations of the tracer data limit the generation of unphysiological profiles. The impact of unphysiological profiles on the selection of the C value is also mitigated due to the prior fits using the mean cohort data. In this way, each cohort has a unique, fixed C value. Identifiability analysis was then repeated at the cohort level using the determined cohort specific C values. The parameter k_2_ was identifiable up to a 95% confidence interval (CI) for 8 of the 16 cohorts, with the remaining cohorts showing only partial/limited identifiability (not to a 95% confidence or not to both the upper and lower 95% thresholds). Limited identifiability was observed for k_a_ for all cohorts.

Identifiability analysis was then performed on the individual mice in each cohort with the results summarised in Table S13 showing the proportion of mice in each cohort for which each parameter was identifiable within a 95% CI. The table shows the elimination rate constant was identifiable for 111/124 mice, with the apparent absorption rate constant identifiable for 42/124. The identifiability of the elimination rate constant is a key result as it is directly included in the IS_P_ calculation. Both k_a_ and k_2_ obtained from tracer fits are shown in Figure S2.

Table S13. Identifiability of k_a_ and k_2_ parameters (with 95% confidence) expressed as a proportion of mice in each cohort.

| Cohort | **k_a_**: Proportion identifiable to 95% confidence | **k_2_**: Proportion identifiable to 95% confidence |
| --- | --- | --- |
| LFAL 4 months | $3/8$ | $8/8$ |
| LFAL 9 months | $2/7$ | $6/7$ |
| LFAL 15 months | $5/8$ | $8/8$ |
| LFAL 21 months | $1/8$ | $7/8$ |
| HFSAL 4 months | $3/8$ | $8/8$ |
| HFSAL 9 months | $5/8$ | $8/8$ |
| HFSAL 15 months | $4/7$ | $7/7$ |
| HFSAL 21 months | $3/8$ | $8/8$ |
| LFDR1 4 months | $3/8$ | $8/8$ |
| LFDR2 9 months | $0/6$ | $5/6$ |
| LFDR1 15 months | $4/8$ | $6/8$ |
| LFDR2 21 months | $0/8$ | $6/8$ |
| HFSDR 4 months | $5/8$ | $8/8$ |
| HFSDR 9 months | $2/8$ | $7/8$ |
| HFSDR 15 months | $2/8$ | $8/8$ |
| HFSDR 21 months | $0/8$ | $3/8$ |

The limited identifiability of k_a_ for specific cohorts was addressed by obtaining a biologically feasible range within which the value of k_a_ is expected to occur. This was achieved by performing 100 000 fits on synthetic data using the mean and standard deviation of the cohort data for each time point. The values obtained for k_a_ were then truncated such that 0 < k_a_ ≤ 0.5, with the 2.5^th^ and 97.5^th^ percentiles used as the lower and upper boundaries, respectively, for each cohort. Consequently, when fitting to individual mouse glucose data, the value for k_a_ would be constrained using these cohort specific ranges. The resulting tracer glucose fits are shown in Figure S.3.

Unlabelled glucose fits were obtained using a customised objective function incorporating the calculated EGP as a source of potential penalty. The objective function was constructed as the sum of 2 parts. Firstly, the sum of squared residuals is calculated:

$${SS}_{Res}=\sum_{i=1}^{n} {({exp}_{i}-{obs}_{i})}^{2}$$

with *exp* the function value, *obs* the specific data value, and *n* the number of data points. Secondly, the EGP (in mM.min^-1^) is calculated for the first 50 minutes of the time course and sampled at 0.1 minute intervals. A value of 1 is added to the absolute of each negative value, with the sum of these values added to SS_Res_. This is then minimised by NMinimize in Mathematica. The negative EGP values were passed as an additional penalty to assist the fitting process by limiting the initial peak, as without this penalty it was observed that some mouse data fits resulted in high initial peaks not considered biologically feasible. These high initial peaks appeared in tandem with negative EGP concentrations in (Vieira-Lara et al., 2023). EGP is not considered a rate that can be negative, as that would imply removal of plasma glucose, which is incorporated in the elimination rate constant, k_2_. As such, it was considered reasonable to penalise the presence of a negative EGP to assist determining the fits. Additionally, the resulting EGP at t=0 minutes was constrained to be larger than EGP at t=10 minutes, as it is expected that EGP will decrease following the administration of a glucose pulse. This approach results in 21/124 EGP profiles with negative values, with the most negative being -0.0176 umol.kg^-1^.min^-1^. These negative values are considered negligible due to the average cohort specific EGP rate during the OGTT being above 100 umol.kg^-1^.min^-1^ (< 0.02%) as shown in Figure 2A.

Parameters obtained during the unlabelled glucose fits are considered phenomenological constants (Vieira-Lara et al., 2023), with the unlabelled k_a_ fixed to the corresponding tracer k_a_ of each mouse to aid in determining biologically pertinent fits. The unlabelled glucose fits are shown in Figure S.4.

Following the tracer and unlabelled glucose fits, the EGP for each mouse was calculated. The EGP rates throughout the time course are shown in Figure S.5 and Figure S6 (umol.kg^-1^.min^-1^ and umol.min^-1^) as the mean for the mice in each cohort with confidence bands as determined by SEM. The plots show the EGP from 0 – 120 minutes, however, average EGP from 5 – 120 minutes was used for average specific EGP calculations (shown in Figure 2A) as well as the IS_L_ (Figure 2B).

The insulin sensitivity indices can then be calculated according to (Vieira-Lara et al., 2023):

$$\text{IS}_{\text{P}}=\frac{\text{k}_{\text{2}}}{\text{INS}_{\text{OGTT}}}$$

$$\text{IS}_{\text{L}}=\frac{\overline{\text{EGP}}\cdot\overline{\text{INS}_{\text{OGTT}}}}{\begin{aligned} \\ \text{ }\text{EGP}\cdot\text{INS}_{\text{OGTT}} \end{aligned}}$$

with IS_P_ the peripheral insulin sensitivity, IS_L_ the liver insulin sensitivity, INS_OGTT_ the cohort average of the time-averaged plasma insulin concentration during the 2 hour time course post glucose administration, EGP the cohort average of the time-averaged specific EGP (from 5-120 minutes). The overlined EGP and INS_OGTT_ are the average of each across all cohorts. The results are shown in the main text Figure 2B.

The decline in IS_P_ with advancing age and an HFS diet is not the result of loss of muscle.

The quadriceps of mice from each cohort were weighed (Figure S7) and indicate that there is not a matching decline in muscle mass in the DR cohorts due to advanced ageing and an HFS diet as seen for the IS_P_ of HFSDR cohorts. Consequently, the mechanism behind the reduced effect of DR in these cohorts remains to be elucidated.

The calculated apparent volumes of distribution resulting from this modelling approach are shown in Figure S8 as normalised to body weight, and in Figure S9 without normalisation to body weight.

Table S14. Sample size for each cohort for each metric/measurement.

| Cohort | **Glucose,**  **k_a_, k_2_,**  **EGP and Volume** | **Quadricep** | **Insulin** | **IS_P_^*^** | **IS_L_^*^** |
| --- | --- | --- | --- | --- | --- |
| LFAL 4 months | 8 | 7 | 8 | 8 | 8 |
| LFAL 9 months | 7 | 7 | 7 | 7 | 7 |
| LFAL 15 months | 8 | 8 | 8 | 8 | 8 |
| LFAL 21 months | 8 | 7 | 8 | 8 | 8 |
| HFSAL 4 months | 8 | 8 | 8 | 8 | 8 |
| HFSAL 9 months | 8 | 8 | 7 | 7 | 8 |
| HFSAL 15 months | 7 | 7 | 8 | 8 | 8 |
| HFSAL 21 months | 8 | 7 | 8 | 8 | 8 |
| LFDR1 4 months | 8 | 7 | 6 | 7 | 7 |
| LFDR2 9 months | 6 | 7 | 0 | - | - |
| LFDR1 15 months | 8 | 7 | 8 | 8 | 8 |
| LFDR2 21 months | 8 | 7 | 8 | 8 | 8 |
| HFSDR 4 months | 8 | 7 | 6 | 6 | 6 |
| HFSDR 9 months | 8 | 8 | 9 | 9 | 9 |
| HFSDR 15 months | 8 | 8 | 8 | 8 | 8 |
| HFSDR 21 months | 8 | 7 | 8 | 8 | 9 |

* Indicates the sample size is theoretical and calculated during application of the propagation of error method.

Comparison of modelling approach

This modelling approach follows strongly from that of Vieira-Lara and colleagues (Vieira-Lara et al., 2023). They determined a fixed C value for all cohorts and subsequently used this for all tracer fits, whereas here we instead determine a fixed C value for each cohort. Additionally, they minimise the sum of squared residuals to obtain the best fits for the unlabelled glucose data, whereas we use negative EGP rates as an additional penalty alongside the sum of squared residuals, with the added constraint that EGP must initially decrease. Consequently, it was determined that a direct comparison to the original approach, may be beneficial. To achieve this, the cohorts reported on in their study were reanalysed using the approach described herein. For brevity, only the IS indices are shown as they incorporate all components of the modelling approach and as such provide a strong indication of any differences in the results. Figure S10 shows both the hepatic, and the peripheral IS determined using both approaches. As can be seen, the hepatic and peripheral IS indices determined using both approaches are nearly identical, with only minor numerical differences. This outcome reinforces the robustness of the results for the control cohorts used in these studies.

Figure S1. Body weights over time for mice included in OGTT experiment. Data shown is the mean of all mice in the respective groups, with the error bars the SEM. Each data point incorporates data from 7 – 32 mice.

Figure S2. Effect of diet and dietary provision on fitted model parameters for apparent glucose absorption and elimination rate constants. Apparent absorption (k_a_, top panel, data shown as discs) and elimination (k_2_, bottom panel, data shown as triangles) rate constants obtained from fitting tracer glucose time course data. Means ± SEM are shown for each cohort. The LFDR cohorts have been separated into 2 subcategories based on the different degree of DR applied. LFDR1: LFDR cohorts receiving 60% of HFSAL calories indicated by the closed symbols at 4- and 15-months. LFDR2: LFDR cohorts receiving 60% of LFAL calories (more restrictive), indicated by the open symbols at 9- and 21-months. n per cohort:

LFAL (4, 15, 21) months = 8, LFAL 9 months = 7. HFSAL (4, 9, 21) months = 8, HFSAL 15 months = 7. LFDR1 (4, 15) months = 8. LFDR2 9 months = 6, LFDR2 21 months = 8. HFSDR (4, 9, 15, 21) months = 8.

Significant ANOVA results for k_2_: p_DR_ = 1.304 x 10^-6^, p_Age_ =3.499 x 10^-11^, p_DRxAge_ =3.552 x 10^-7^, p_DietxAge_ = 3.064 x 10^-5^, p_DRxDietxAge_ = 0.004624.

Figure S3. Tracer glucose fits for each cohort (age and diet paired). The coloured bands indicate the average ± SEM of the individual mouse fits for each cohort. Mean for each timepoint ± SEM are also shown as the datapoints and error bars respectively. n per cohort:

LFAL (4, 15, 21) months = 8, LFAL 9 months = 7. HFSAL (4, 9, 21) months = 8, HFSAL 15 months = 7. LFDR1 (4, 15) months = 8. LFDR2 9 months = 6, LFDR2 21 months = 8. HFSDR (4, 9, 15, 21) months = 8.

Significant ANOVA results for peak tracer concentration:

p_DR_ = 4.440 x10^-11^, p_Diet_ = 6.052 x10^-9^, p_Age_ = 6.416 x10^-8^.

Figure S4. Unlabelled glucose fits for each cohort (age and diet paired). Coloured bands indicate the average ± SEM of the mouse fits. Mean for each timepoint ± SEM are shown as the datapoints and error bars respectively. n per cohort:

LFAL (4, 15, 21) months = 8, LFAL 9 months = 7. HFSAL (4, 9, 21) months = 8, HFSAL 15 months = 7. LFDR1 (4, 15) months = 8. LFDR2 9 months = 6, LFDR2 21 months = 8. HFSDR (4, 9, 15, 21) months = 8.

Figure S5. Age and diet paired cohort specific EGP (normalised to BW) time courses. The coloured bands indicate the average ± SEM of the individual mouse EGP time courses in each cohort. n per cohort:

EGP: LFAL (4, 15, 21) months = 8, LFAL 9 months = 7. HFSAL (4, 9, 21) months = 8, HFSAL 15 months = 7. LFDR1 (4, 15) months = 8. LFDR2 9 months = 6, LFDR2 21 months = 8. HFSDR (4, 9, 15, 21) months = 8.

Significant ART-ANOVA results for steady-state specific EGP:

p_DR_ = 0.00084144, p_Diet_ = 0.00183543, p_DietxAge_ = 0.03077719.

Figure S6. Age and diet paired cohort EGP time courses not normalised to BW. The coloured bands indicate the average ± SEM of the individual mouse EGP time courses in each cohort. LFDR1 cohorts are annotated as DR1 and LFDR2 cohorts annotated as DR2 with the time-courses shown as dashed lines. n per cohort:
LFAL (4, 15, 21) months = 8, LFAL 9 months = 7. HFSAL (4, 9, 21) months = 8, HFSAL 15 months = 7. LFDR1 (4, 15) months = 8. LFDR2 9 months = 6, LFDR2 21 months = 8. HFSDR (4, 9, 15, 21) months = 8.

Significant ANOVA results for time-averaged EGP not normalised to BW:

p_DR_ = 7.452 x 10^-9^, p_Diet_ = 0.0104636, p_Age_ = 0.0004068, p_DietxAge_ = 0.0143884.

Significant ANOVA results for steady-state EGP not normalised to BW:

p_DR_ = 2.972 x 10^-9^, p_Diet_ = 0.0110855, p_Age_ = 0.0002709, p_DietxAge_ = 0.0017708.

Figure S7. Mouse average quadriceps mass. Mean quadriceps mass (data shown as discs) ± SEM for each cohort. Data shown for 6-month cohorts are from the quadriceps of 1 leg, not the mean of 2 legs. LFDR2 cohorts (receiving 60% of LFAL calories i.e. more restricted than LFDR1 cohorts) are indicated by the open symbols. n per cohort:
LFAL (4, 9, 21) months = 7, LFAL 15 months = 8. HFSAL (4, 9) months = 8, HFSAL (15, 21) months = 7. LFDR1 (4, 15) months = 7, LFDR2 (9, 21) months = 7. HFSDR (4, 21) = 7, HFSDR (9, 15) = 8.

Figure S8. Apparent volume of distribution (normalised to BW). Mean apparent volume of distribution normalised to BW ± SEM. LFDR2 cohorts are indicated by the open symbols. n per cohort:
LFAL (4, 15, 21) months = 8, LFAL 9 months = 7. HFSAL (4, 9, 21) months = 8, HFSAL 15 months = 7. LFDR1 (4, 15) months = 8. LFDR2 9 months = 6, LFDR2 21 months = 8. HFSDR (4, 9, 15, 21) months = 8.

Significant ANOVA results:

p_DR_ < 2.2 x 10^-16^, p_Diet_ < 2.2 x 10^-16^, p_Age_ < 2.2 x 10^-16^, p_DRxDiet_ = 0.01729, p_DRxAge_ = 5.597 x 10^-12^, p_DietxAge_ = 1.5 x 10^-12^.

Figure S9. Apparent volume of distribution (not normalised to BW). Mean apparent volume of distribution (not normalised to BW) ± SEM. LFDR2 cohorts are indicated by the open symbols. n per cohort:

LFAL (4, 15, 21) months = 8, LFAL 9 months = 7. HFSAL (4, 9, 21) months = 8, HFSAL 15 months = 7. LFDR1 (4, 15) months = 8. LFDR2 9 months = 6, LFDR2 21 months = 8. HFSDR (4, 9, 15, 21) months = 8.

Significant ANOVA results:

p_DR_ = 0.002064, p_Age_ < 2.2 x 10^-16^, p_DRxAge_ = 2.014 x 10^-6^, p_DietxAge_ = 6.824 x 10^-5^, p_DRxDietxAge_ = 0.028680

Figure S10. IS_L_ (top panel, data shown as discs) and IS_P_ (bottom panel, data shown as triangles) for the control cohorts analysed by Vieira-Lara and colleagues (Vieira-Lara et al., 2023) using their original modelling approach and this adapted approach. IS_L_ is dimensionless as it is normalised to the cohorts of the study. Calculated sample size for each cohort:
Original IS_L_: LFAL 4 months = 9, LFAL (9, 15) months = 7, LFAL 21 months = 8. HFSAL (4, 9) months = 7, HFSAL 15 months = 8, HFSAL 21 months = 6.
Adapted IS_L_: LFAL (4, 15, 21) months = 8, LFAL 9 months = 7. HFSAL (4, 9, 15, 21) months = 8.

Original and adapted IS_P_: LFAL (4, 15, 21) months = 8, LFAL 9 months = 7. HFSAL (4, 15, 21) months = 8, HFSAL 9 months = 7.
